# Targeting SHP2 with an Active Site Inhibitor Blocks Signaling and Breast Cancer Cell Phenotypes

**DOI:** 10.1021/acsbiomedchemau.3c00024

**Published:** 2023-07-14

**Authors:** Dhanaji M. Lade, Yehenew M. Agazie

**Affiliations:** One Medical Center Drive, Department of Biochemistry and Molecular Medicine, School of Medicine, West Virginia University, P.O. Box 9142, Morgantown, West Virginia 26506, United States

**Keywords:** SHP2, inhibitors, CNBCA, signaling, breast cancer

## Abstract

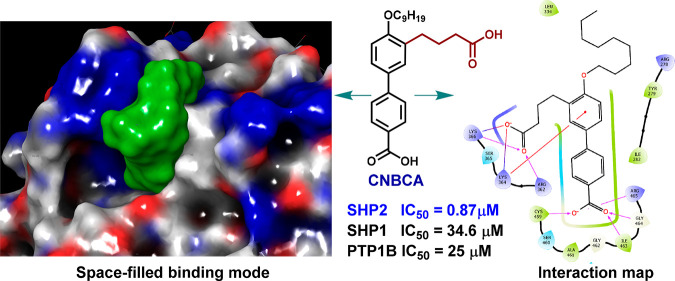

The Src homology phosphotyrosyl phosphatase 2 (SHP2) is an oncogenic protein for which targeted therapies are being sought. In line with this idea, we have previously reported the development of a specific active site inhibitor named CNBDA that showed effectivity in suppressing the transformation phenotypes of breast cancer cells. To improve efficacy, we introduced limited modifications to the parent compound and tested potency *in vitro* and under cell culture conditions. Of these modifications, removal of one of the butyric acid groups led to the production of a compound named CNBCA, which showed a 5.7-fold better potency against the SHP2 enzyme activity *in vitro*. In addition, CNBCA showed better selectivity to SHP2 than the control PTPs (SHP1 and PTP1B) as determined by the phosphatase assay. Furthermore, CNBCA binds and inhibits enzyme activity of full-length SHP2 in cellular contexts, downregulates SHP2 mediated signaling, and suppresses breast cancer cell phenotypes, including cell proliferation, colony formation, and mammosphere growth. These findings show that targeting SHP2 with CNBCA is effective against the cancerous properties of breast cancer cells.

## Introduction

The Src homology phosphotyrosyl phosphatase 2 (SHP2) is a positive regulator of receptor tyrosine kinase (RTK) signaling,^1−6^ and by doing so it promotes the cancerous phenotype of tumor cells, including breast cancer.^7,8^ For instance, SHP2 promotes the transformation phenotypes of the HER2-positive (HER2+) and the triple-negative breast cancer (TNBC) cells, including epithelial to mesenchymal transition in 2D culture, colony formation in soft agar, mammosphere formation in suspension cultures, extracellular matrix degradation and invasion in 3D matrigel, and tumorigenesis *in vivo*.^7−11^

SHP2 is a 595 amino acid long cytoplasmic phosphotyrosyl phosphatase (PTP) encoded by the PTPN11 gene (NCBI accession ID: XP_054228694.1). The SHP2 protein is composed of two SH2 domains in the N-terminal and a phosphotyrosyl phosphatase (PTP) domain in the C-terminal regions.^12^ The SH2 domains play an adaptor role by mediating interaction with phosphotyrosine (pTyr) on signaling proteins that function in the RTK signaling pathway. This allows the PTP domain to access tyrosine phosphorylated biological substrates and catalyze dephosphorylation reactions.^13,14^ How a tyrosine phosphatase becomes a requisite signal transducer in a signaling pathway that depends on tyrosine phosphorylation has not been fully understood. Available literature suggests that SHP2 specifically dephosphorylates negative regulatory pTyr sites in RTKs and downstream signaling proteins to augment and sustain downstream signaling. For instance, SHP2 dephosphorylates RasGAP (Ras GTPase) docking sites on EGFR (pTyr992) and on HER2 (pTyr1023) to sustain the activated form of Ras (GTP-Ras).^2,15^ SHP2 has also been shown to dephosphorylate pTyr397 in FAK to facilitate focal adhesion turnover^16^ and pTyr314 in PAG to enhance Src Tyr kinase signaling.^17^ The positive role of SHP2 in tyrosine kinase signaling in general has attracted interest to develop specific inhibitors for use in diseases, particularly in cancer, that often involve hyperactive tyrosine kinase signaling.

The oncogenic property of SHP2 has spurred much interest for developing targeted therapies in cancer. As such, significant efforts have been devoted to developing specific inhibitors. Initial efforts primarily focused on developing active-site SHP2 inhibitors (ASSIs) that competitively inhibit PTPase (enzyme) activity. Notable active site inhibitors that have been reported and used by other investigators for inhibiting SHP2 include: PHPS1,^18^ SPI-112Me,^19^ II-B08,^20^ 11a-1,^21^ 220-32,^22^ compound 45,^23^ and our compound CNBDA.^24^ Relatively recently, inhibitors that bind to allosteric clefts in SHP2 have been reported, including SHP099 and its derivatives,^25,26^ and IACS-15414.^27^ The difference between the two classes of inhibitors is that the ASSIs bind to the open and activated SHP2, while the allosteric inhibitors bind to the closed and inactive SHP2. Despite these concerted efforts, no SHP2 targeting drugs have reached the clinic yet.

Since activation of SHP2 involves its interaction with pTyr via the two SH2 domains, the abundant form of the SHP2 molecules in cancers with hyperactive Tyr kinase signaling is likely to be in an open and active conformation. As such, active-site inhibitors are likely to be more effective in cancers with hyperactive Tyr kinase signaling since they bind to the open and active SHP2. In line with this concept, we have recently reported an active site SHP2 inhibitor named CNBDA that showed promising antibreast cancer cell effects.^24^ In the current study, we report modifications to CNBDA, which enhanced its antibreast cancer cell effects.

## Results and Discussion

### Design and Molecular Docking

**Figure 1:**
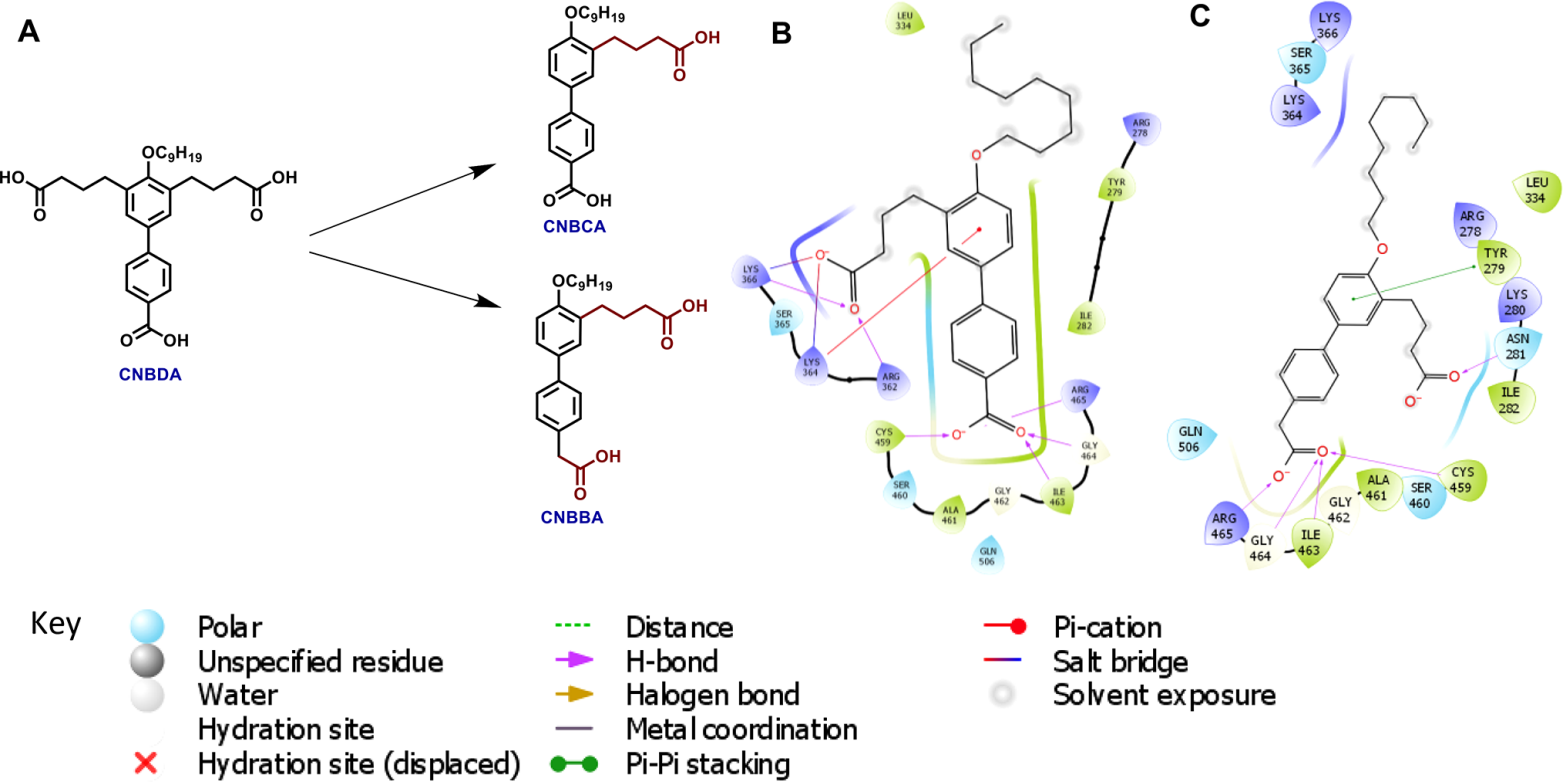
(A) Structures of CNBDA, CNBCA, and CNBBA. (B) Interaction map of CNBCA to the SHP2 active site in the PTP domain structure (ID: PDB 4PVG). (C) Interaction map of CNBBA to the SHP2 active site in the PTP domain structure (ID: PDB 4PVG).

We have previously reported the design, synthesis, and characterization of a novel active site SHP2 inhibitor named CNBDA that has three polar carboxylic groups.^24^ Although CNBDA showed effectiveness in inhibiting the SHP2 enzyme activity under *in vitro* conditions, the presence of three polar groups makes it less useful for future *in vivo* studies. We thus made two modifications that reduce the number of carboxylic groups from three to two. Furthermore, we have replaced one of the carboxylic groups with an acetic acid group in one of the modified structures. By doing so, we designed two derivatives, which are named CNBCA (3′-(3-carboxypropyl)-4′-(nonyloxy)-[1,1′-biphenyl]-4-carboxylic acid) and CNBBA (4-(4′-(carboxymethyl)-4-(nonyloxy)-[1,1′-biphenyl]-3-yl) butanoic acid). The aliphatic nonane group that was included in the parent compound to enhance cellular uptake was maintained in both CNBCA and CNBBA. The structures of the parent compound CNBDA and the two derivatives are shown in Figure 1A. We first used *in silico* molecular docking to predict similarities and differences in interaction properties between the parent compound CNBDA and the two newly designed compounds. We used the molecular modeling program Glide (Schrodinger) with induced-fit docking and binding energy calculation capabilities for these predictions.^28^ Each molecule was docked into the SHP2 active site, using the crystal structure of the PTP domain (PDB: 4PVG) that was solved in complex with the active site inhibitor 11a-1.^21^ The docking results showed CNBCA making nine interactions with the SHP2 active site mediated by H-bonding, π–cation, and ionic interactions, while CNBBA making six interactions mediated by H-bonding, ionic interaction, and pi-pi stacking (Figure 1B and C). As shown, the aliphatic nonane group is exposed to the solvent, suggesting minimal participation in the binding of CNBCA and CNBBA to the active site. We also docked each modified compound to the SHP1 active site (PDB: 1GWZ),^29^ the close structural homologue of SHP2, for the purpose of comparison. SHP1 is a 597 amino acid protein encoded by the PTPN6 gene (NCBI accession ID: XP_054228689.1). The docking data predict very few interactions with the SHP1 active site (Supporting Figure 1), suggesting that the two derivatives make stronger interactions with the SHP2 active site but very weak interactions with the SHP1 active site. They also suggest that the interaction of CNBCA to the SHP2 active site is comparable to that of the parent compound CNBDA, which also made nine similar interactions.^24^

### Chemistry

As outlined in the synthesis scheme shown below, the synthesis of CNBCA began with Suzuki cross-coupling reaction between 5-bromosalicylaldehyde (**1**) and 4-cyanophenylboronic acid (**2**) using Pd(PPh_3_)_4_ (5 mol %) and Na_2_CO_3_ in PhCH_3_:MeOH:H_2_O heated at 80 °C for 12 h to furnish **3a** in 86% yield. Further, the Wittig olefination reaction was optimized using (2-cyanoethyl) triphenylphosphonium bromide, which was unstable with other bases. When three carbon-containing (2-cyanoethyl) triphenylphosphonium bromide salt was treated with K_2_CO_3_ in combination with one equivalent of water in THF, it generated stable Witting ylide. Addition of aldehyde **3a** to that and stirring at room temperature for 16 h provided olefin **4a** in 50% yield. Next, the double bond was reduced using Pd–C/H_2_ to furnish saturated adduct **5a** in 90% yield. This was O-alkylated using 1-bromononane to obtain **6a** with 75% yield. At the end, both nitrile groups were hydrolyzed using NaOH in ethanol to furnish CNBCA in 74% yield (see the scheme below).

For the synthesis of CNBBA, 5-bromosalicylaldehyde (**1**) was reacted with 4-(4,4,5,5-tetramethyl-1,3,2-dioxaborolan-2-yl) benzeneacetonitrile (**2b**) under Suzuki cross-coupling conditions to give **3b** at 82% yield. Next, a similar synthetic sequence to that of CNBCA was followed (hydrogenation, O-alkylation, hydrolysis) to obtain CNBBA with 75% yield. The formation of CNBCA and CNBBA was confirmed by NMR and mass spectroscopy analysis. The spectral data and ^1^H and ^13^C NMR information are provided in the Supporting Information.

#### Synthesis Scheme

**Scheme 1:**
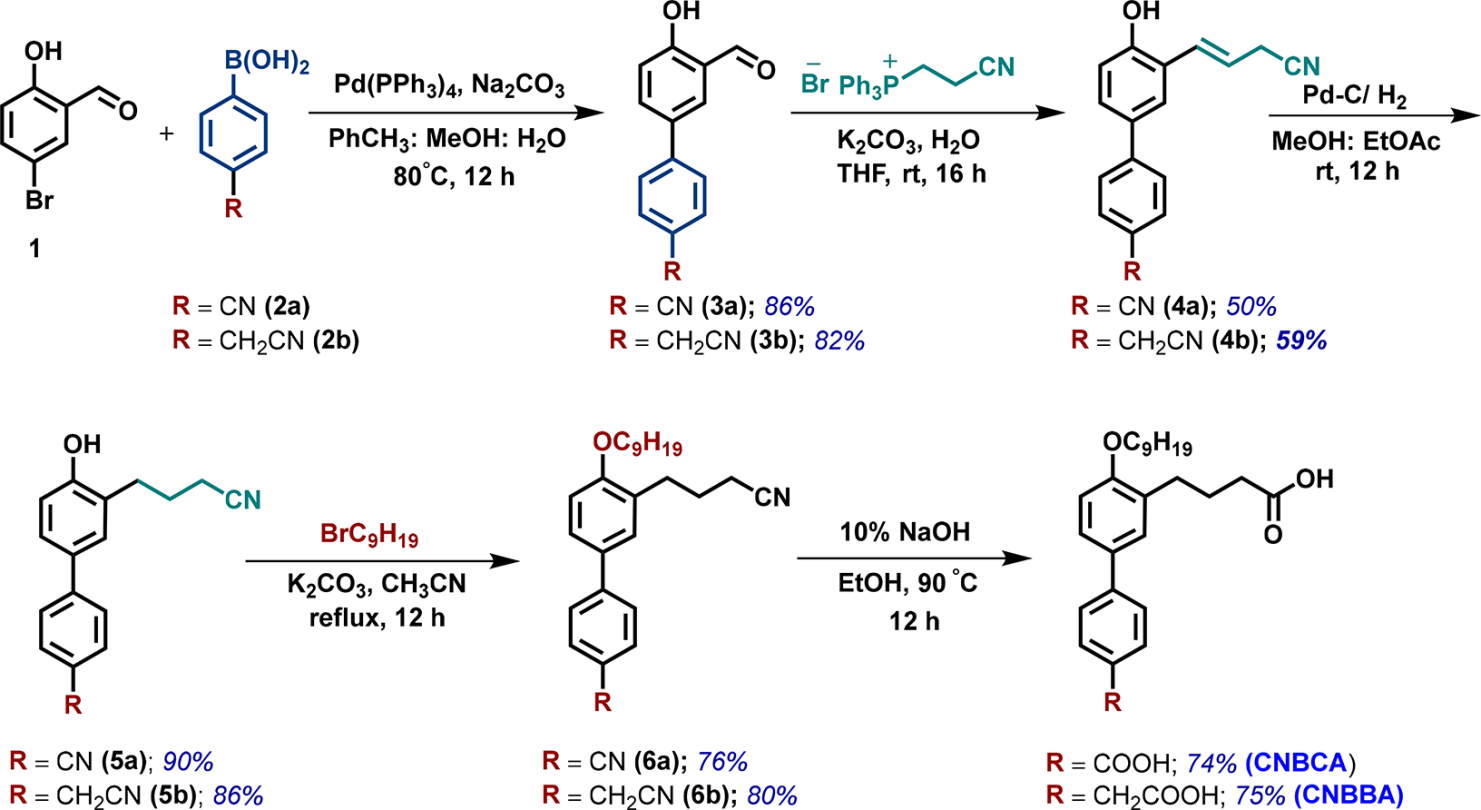


The synthesis scheme for CNBCA and CNBBA can be found in Scheme 1.

### CNBCA and CNBBA Inhibit the SHP2 Enzyme Activity

After completion of synthesis, both CNBCA and CNBBA were purified on silica gel, and purity was determined by HPLC. The purified compounds were then tested for their effect on the SHP2 enzyme activity and on the activity of the selected control PTPs (SHP1 and PTP1B) by the phosphotyrosyl phosphatase (PTPase) assay as described previously.^30,31^ First, the GST fusion of the PTP domains of SHP2, SHP1, and PTP1B were produced and purified as reported by us previously.^32^ The reactions were performed in a buffer containing 10 mM Tris-HCl (pH 7.2), 100 mM NaCl, 1 mM EDTA, 1 mM dithiothreitol (DTT), and 0.01% Tween-20. Briefly, CNBCA or CNBBA was added first in a serial dilution ranging from 100 μM to 97 nM followed by the enzymes (SHP2, SHP1, or PTP1B) to a final concentration of 1 nM. After 5 min of incubation at room temperature, the reactions were started by adding the artificial substrate DiFMUP (6,8-difluoro-4-methylumbelliferyl phosphate) to a final concentration of 20 μM for SHP2, 35 μM for SHP1, and 10 μM for PTP1B in a reaction volume of 100 μL; variations in DiFMUP concentrations reflect the reported Km values for the respective PTPs.^33^ The complete mixture was incubated at 37 °C for 20 min, and fluorescence intensity was measured by the Synergy 4 plate reader at the excitation and emission wavelengths of 360 and 460 nm, respectively. The half inhibitory concentration (IC_50_) value of each compound was calculated and plotted using Graphpad Prism software. The results showed inhibition of the SHP2 enzyme activity by CNBCA with an IC_50_ of 0.87 μM and by CNBBA with an IC_50_ of 5.1 μM (Table 1). While CNBCA showed significant improvement in potency over that of the parent compound (0.87 μM versus 5.0 μM), CNBBA exhibited similar potency as that of the parent compound (5.1 μM versus 5.0 μM). Comparative fold difference calculations showed that CNBCA is better than the parent compound CNBDA and the other new compound (CNBBA) by 5.7 fold and 5.8 fold, respectively.

**Table 1:** Inhibitory Effect of CNBCA and CNBBA on the Enzyme Activity of SHP2^a^

compd	SHP2 IC_50_, μM	fold difference
CNBCA	*0.87*	*1.0*
CNBBA	5.1	5.8
CNBDA	5.0	5.7

a Included is the IC_50_ of the previously published parent compound for the purpose of comparison.

We have previously shown that the parent compound CNBDA is selective for inhibition of SHP2 over that of SHP1 by approximately 25 fold.^24^ To test whether the relatively potent inhibitor CNBCA is also selective, we conducted comparative inhibition studies against the close structural homologue SHP1 and the ubiquitously expressed PTP1B, using the PTPase assay. The GST fusion PTP domains of SHP1 and PTP1B were expressed and purified in a fashion similar to that of SHP2. The results showed less effectiveness of CNBCA against SHP1 and PTP1B as evidenced by the significantly higher IC_50_ values, 34.6 μM and 25.0 μM, respectively (Table 2). Calculation of selectivity ratios using the SHP2 IC_50_ as a reference showed that CNBCA is more selective to SHP2 than for SHP1 and PTP1B by 39.77-fold and 28.73 fold, respectively. Taken together, data in Table 1 and Table 2 suggest that CNBCA is more potent and selective than parent compound CNBDA.

**Table 2:** Comparative IC_50_ Values of the Most Potent Derivative CNBCA against SHP2 and two other PTPs, SHP1 and PTP1B, the Close Structural Homologue and the Most Ubiquitous PTP, Respectively

PTP	IC_50,_ μM	selectivity ratio
SHP2	*0.87*	1.0
SHP1	34.6	39.77
PTP1B	25.0	28.73

### CNBCA is a Competitive SHP2 Inhibitor

**Figure 2:**
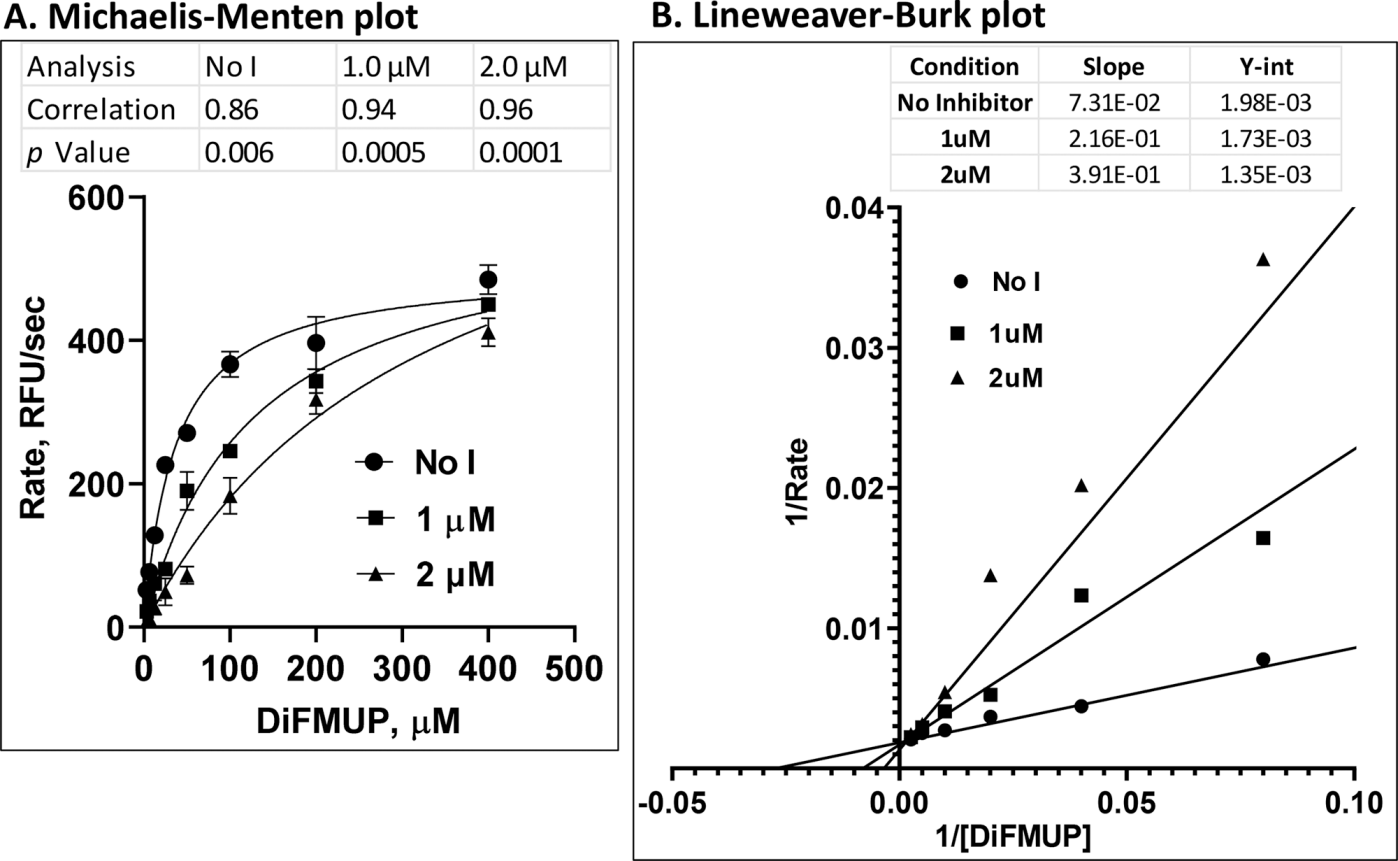
(A) Michaelis–Menten plot showing a shift in DiFMUP concentration (*K*_m_) without a change in *V*_max_ when CNBCA is added at 1.0 and 2.0 μM concentration. The results are mean ± SD of three independent experiments. The table above the graph shows Pearson Correlation coefficients (*r*) and *p* values of X values versus every Y data set. (B) Lineweaver–Burk plot, confirming an increase in the *Km* values without affecting the enzyme rate. The table above the graph shows the slope and the Y intercepts.

As suggested by the findings of the molecular docking studies (Figure 1), CNBCA is designed to bind to the SHP2 active site. As such, its mechanism of inhibition is expected to be through competitive binding to the active site. To verify this point, we conducted enzyme kinetic studies using the artificial substrate DiFMUP in the presence and absence of CNBCA. Briefly, 400 μM DiFMUP in the PTPase buffer was added to the 12th well in the 96-well plate and then serially diluted to as low as 3.12 μM in the successive wells. After adding the enzyme to a final concentration of 1.0 nM in a total volume of 100 μL, the fluorescence of the enzyme reaction rate was measured for a total of 3 min at 10 s interval in the Synergy 4 plate reader at the excitation and emission wavelengths of 360 and 460 nm, respectively. The data was transformed into relative fluorescence unit (RFU) per second and plotted using the Graphpad software to produce the Michaelis–Menten and the Lineweaver–Burk plots. The Michaelis–Menten plot showed the dependence of the enzyme rate on substrate concentration, which was maximized as the DiFMUP concentration became saturating (Figure 2A). Addition of CNBCA at 1.0 μM concentration shifted the rate to the right, and this effect was enhanced by increasing the amount to 2.0 μM, suggesting the occurrence of competitive inhibition. Calculation of the Pearson correlation coefficients (*r*) showed the significance of the findings. A Lineweaver–Burk or double-reciprocal plot of the same data showed a shift to the left (increased DiFMUP) to achieve maximum catalytic rate or *V*_max_ following the addition of CNBCA (Figure 2B). Accordingly, the calculated *K*_m_ values were 36.05, 124.6, and 290.6 μM in the absence and in the presence of 1.0 and 2.0 μM CNBCA, respectively. Overall, data in Figure 2 show that CNBCA is a competitive inhibitor.

### CNBCA Binds to Full-Length SHP2 and Inhibits Enzyme Activity

Since the PTPase data described in Tables 1 and 2, and Figure 2 were performed using isolated PTP domains lacking the regulatory SH2 domains, it was necessary to demonstrate whether CNBCA is also effective against full-length SHP2 (FL-SHP2). First, we used the cellular thermal shift assay (CETSA) to show whether CNBCA binds to endogenous full-length SHP2 under cellular contexts. The CETSA assay is based on the principle that specific inhibitors bind to a target protein and confer relative stability from heat-induced denaturation and precipitation when compared to a nontarget protein present in the same milieu.^34^ Accordingly, we performed CETSA to test the effect of CNBCA on the SHP2 protein stability and compared it with the nontarget PTP1B protein as described in the materials and methods.

**Figure 3:**
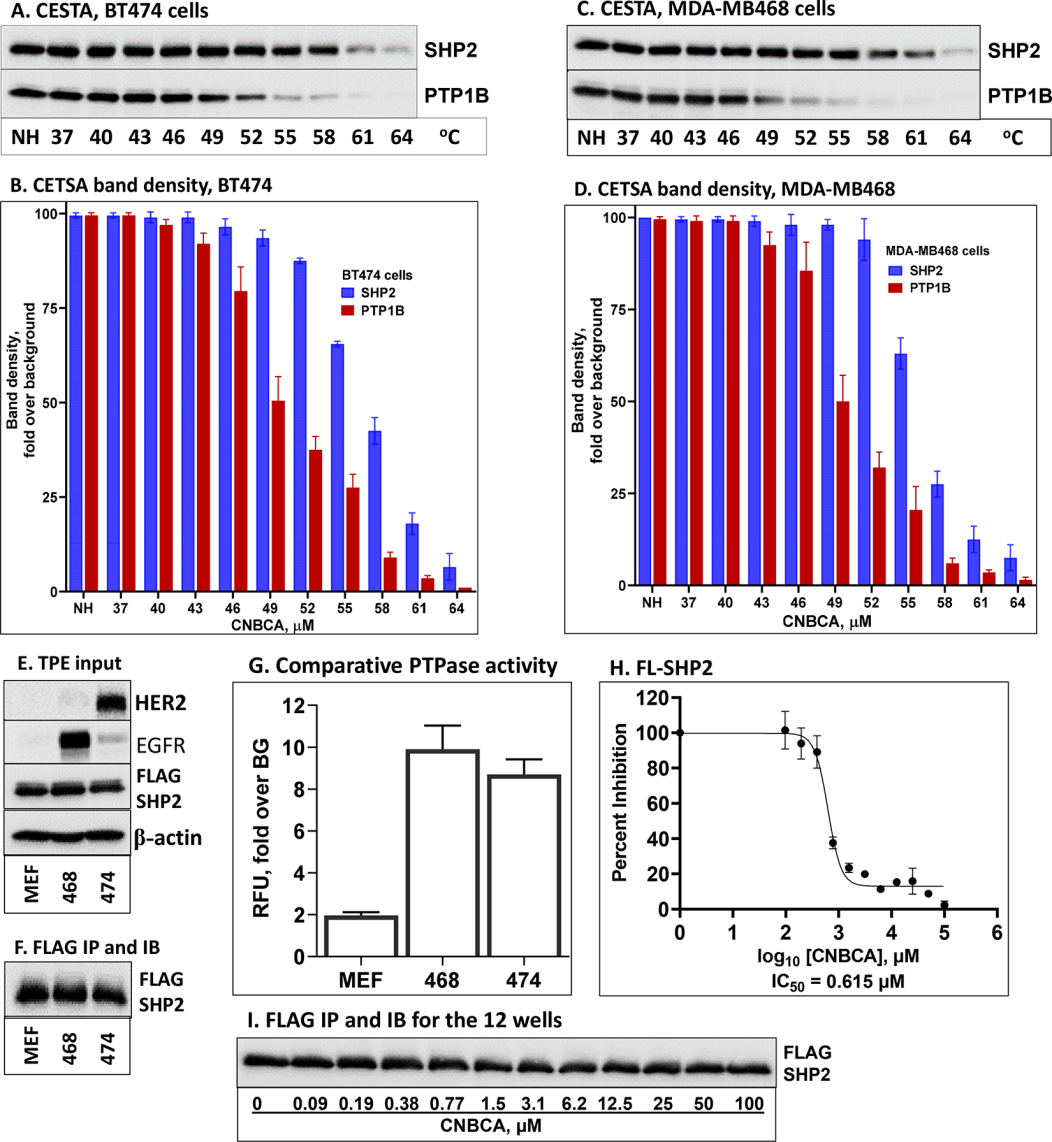
Cellular thermal shift assay (CETSA). (A) IB analysis of BT474 lysates treated with 100 μM CNBCA. (C) IB analysis of MDA-MB468 cell lysates treated with CNBCA. (B) Bar graph showing the SHP2 and the PTP1B band densities of the IB data from BT474 cell lysates. (C) JIMT-1 cell lysates treated with BPDA2. (D) . Bar graph showing the SHP2 and PTP1B band densities of the IB data from MDA-MB468 cell lysates. The bar graphs were plotted using the Mean ± SD of band densities from three independent experiments. (E) IB analysis total protein extract (TPE) for expression of FLAG-tagged FL-SHP2 and endogenous EGFR and HER2. (F) IB analysis of FL-SHP2 immunoprecipitated with anti-FLAG antibody. (G) Comparative PTPase assay for activity of FL-SHP2 expressed in MEFs, MDA-MB468, and BT474 cells. The fold relative fluorescence unit (RFU) was calculated by dividing by the background (BG) signal. (H) Line graph showing the PTPase assay for determining the effect of CNBCA on FL-SHP2. (I) IB analysis of SHP2 immunoprecipitates for the 12 wells used for the PTPase assay in panel H.

After heat treatment, supernatants were analyzed by immunoblotting (IB) for SHP2 and the nontarget phosphatase PTP1B. The results showed stabilization of SHP2 in solution by CNBCA treatment, but this effect was not as robust for PTP1B as evidenced by differences in signal intensity as the temperature of heat treatment increased (Figure 3A and C). To provide semiquantitative data, band densities of both SHP2 and PTP1B were measured and presented as bar graph. The results showed stabilization of SHP2 in solution to nearly 100% at 49 °C, to 95% at 52 °C, to 70% at 55 °C, and to 45% at 58 °C. On the other hand, the nontarget protein PTP1B was reduced to approximately 50% at 49 °C, to 25% at 55 °C, and to less than 10% at 58 °C (Figure 3B and D). These findings clearly show that CNBCA differentially stabilizes SHP2 in solution by binding to the full-length protein (FL-SHP2). On the other hand, CNBCA is less effective in stabilizing the nontarget protein PTP1B, suggesting its specificity to SHP2.

To show whether CNBCA also inhibits cellular SHP2, we first compared the enzyme activity of FL-SHP2 expressed in fibroblasts and BC cells as FLAG-tagged protein.^35^ To confirm efficiency of the expression, we conducted IB analysis of total protein extracts (TPE) prepared from the noncancerous MEF cells (mouse embryo fibroblasts), the MDA-MB468 BC cells that overexpress EGFR, and the BT474 BC cells that overexpress HER2. The results showed the efficient expression of FL-SHP2 as determined by anti-FLAG IB (Figure 3E). We also performed HER2 and EGFR IB analyses, which confirmed overexpression of these RTKs in the BC cells, but not in the MEF cells. IB for β-actin was used as a loading control, which appears to be comparable in all lanes. Next, FL-SHP2 was immunoprecipitated (IP) with anti-FLAG antibody from the TPEs of the three cells (Figure 3F) and used for determining the activation state while still bound to protein-A sepharose beads, as described in the materials and methods. As shown in Figure 3G, SHP2 expressed in BC cells was enzymatically more active by at least 5 fold than SHP2 expressed in the noncancerous MEF cells, suggesting that hyperactive Tyr kinase signaling activates the PTPase function of SHP2. We thus used SHP2 expressed in BC cells to test the inhibitory effect of CNBCA on FL-SHP2. Accordingly, FL-SHP2 was immunoprecipitated from the TPE of the MDA-MB468 cells with anti-FLAG antibody, divided equally into 12 wells, treated with CNBCA in a serial dilution, ranging from 95 nM to 100 μM, incubated at room temperature for 5 min, and then tested by a PTPase assay as described above for the PTP domain. The results showed inhibition of FL-SHP2 by CNBCA with an IC_50_ of 0.615 μM (Figure 3H), which is slightly better than the IC_50_ observed with the PTP domain (see Table 1). IB analysis of immunoprecipitates from each well showed comparable protein levels in all lanes (Figure 3I). However, it was not possible to estimate the concentration of FL-SHP2 in each well due to the nature of the experiment. Nonetheless, these findings suggest that CNBCA is also effective against FL-SHP2, complementing the CETSA results.

### CNBCA Downregulates Basal Signaling in Breast Cancer Cells

**Figure 4:**
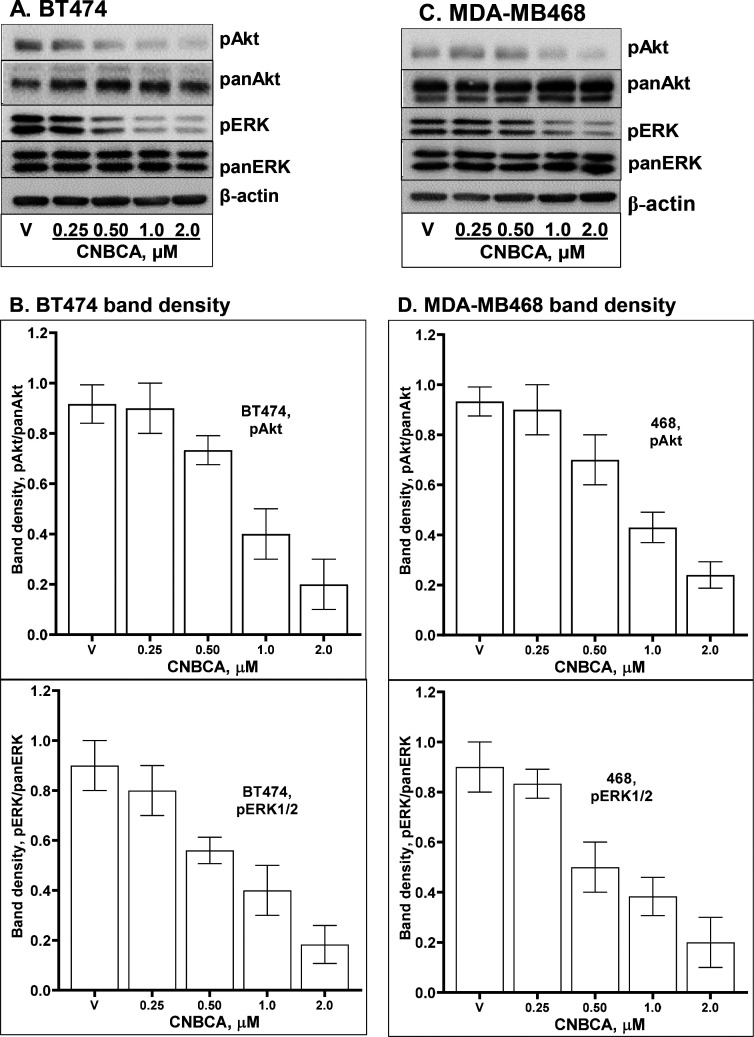
Effect of CNBCA on basal signaling. (A) IB image data showing the effect of CNBCA on basal activation of Akt (pAkt) and ERK1/2 (pERK1/2) in the BT474 BC cells. (B) Band density measurement of pAkt and pERK1/2 in the BT474 cells presented as the ratio of activated over total protein. (C) IB image data showing effect of CNBCA on basal activation of Akt (pAkt) and ERK1/2 (pERK1/2) in the MDA-MB468 BC cells. (D) Band density measurement of pAkt and pERK1/2 in the MDA-MB468 cells presented as ratio of activated over total protein. Data are presented as Mean ± SD.

Since SHP2 is an essential mediator of mitogenic and cell survival signaling in breast cancer cells, we determined the effect of CNBCA treatment on activation of ERK1/2 and Akt in the HER2-positive BT474 and in the triple-negative MDA-MB468 cell lines. Cells growing in 2D culture were treated with varying concentrations of CNBCA, ranging from 0.250 to 2.0 μM, for 48 h, and total cell lysates were analyzed by immunoblotting (IB) with antibodies that recognize the activated forms of ERK1/2 (pERK1/2) and Akt (pAkt). The results showed downregulation of basal pERK1/2 and pAkt levels by CNBCA treatment in a concentration dependent manner (Figure 4A and C). IB analysis for total Akt (panAkt), total ERK1/2 (panERK), and β-actin showed similar total protein levels in all lanes. Band density measurements from three independent experiments showed that CNBCA inhibited SHP2 mediated Akt and ERK1/2 activation in the BT474 and MDA-MB468 cells with an approximate IC_50_ of 1.0 μM (Figure 4B and D). These findings suggest that CNBCA effectively downregulates SHP2 mediated signaling in BC cells.

### CNBCA Suppresses BC Cell Phenotypes

**Figure 5:**
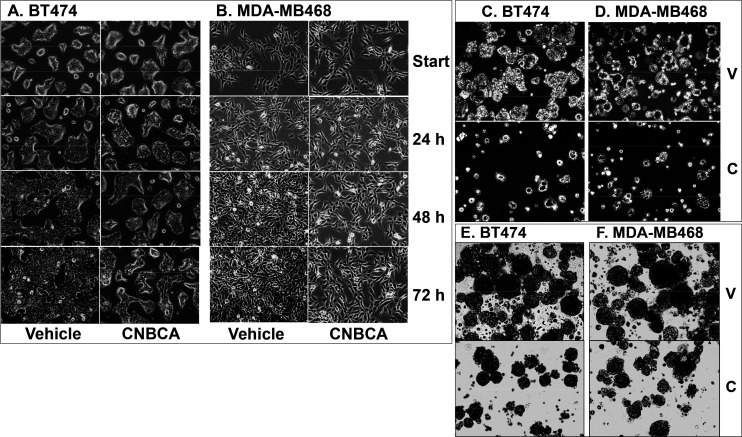
Effect of CNBCA on the growth of BT474 (A) and MDA-MB468 (B) breast cancer cells in a 2D culture. Effect of CNBCA on colony formation by BT474 (C) and MDA-MB468 (D) breast cancer cells in soft agar. Effect of CNBCA on mammosphere formation by BT474 (E) and MDA-MB468 (F) breast cancer cells in suspension culture. V: Vehicle; C: CNBCA.

Since SHP2 is essential for the growth and transformation of BC cells,^8,9,11^ we determined the effect of SHP2 inhibition with CNBCA in 2D and 3D culture systems. For the effect on cell growth, BC cells (BT474 and MDA-MB468) were seeded in 2D culture and treated with a single concentration of CNBCA (500 nM), the concentration that provided a 50% inhibition of SHP2 mediated signaling in BT474 cells. Images were collected at the start and every 24 h for a total of 72 h (3 days). The results showed growth of the vehicle treated cells to confluency during this period, but the CNBCA treated cells were still half-confluent, suggesting suppression of cell proliferation (Figure 5A and B). For effect on anchorage independent growth, approximately 10^6^ cells were seeded in soft agar as described previously,^24^ and treated with CNBCA at 500 nM concentration for 10 days with refeeding and adjustment of compound concentration. Images collected at the 4× microscopic objective showed that the vehicle treated cells formed more and larger colonies, while the CNBCA treated cells formed fewer and smaller colonies (Figure 5C and D). Finally, the effect of CNBCA on cancer stem cell properties was determined by the suspension assay, in which only cells with stem-like properties only can grow and form mammospheres.^7,24,36^ Approximately 10^6^ cells were seeded in nonadherent 6 cm plates and treated with CNBCA (500 nM) for 10 days with complementation of media and drug concentration. The results showed formation of larger mammospheres by the vehicle treated cells and fewer and smaller mammospheres by the CNBCA treated cells (Figure 5E and F). These finding suggest that CNBCA is effective in suppressing the cancer stem cell properties of BC cells.

## Conclusions

In this study, we report modification of the parent active site SHP2 inhibitor CNBDA to produce derivatives with improved potency. Of the two derivatives, CNBCA showed significantly improved potency in inhibiting the PTPase activity of SHP2. More specifically, CNBCA inhibited the SHP2 PTPase activity with an IC_50_ of 0.87 μM, which is better than the parent compound that inhibited the SHP2 PTPase activity with an IC_50_ of 5.0 μM. In addition, the PTPase assay data showed that CNBCA is more selective to SHP2 than the control PTPs (SHP1 and PTP1B), and is a competitive inhibitor. Furthermore, CNBCA binds to FL-SHP2 and inhibits its enzyme activity, downregulates SHP2 mediated signaling, and suppresses the growth and transformation phenotypes of breast cancer cells. Overall, data reported here show that CNBCA that lacks one of the butyric acid groups of the parent compound functions with better potency in both inhibiting SHP2 enzyme activity and suppressing breast cancer cell phenotypes.

## Experimental Section

### Molecular Docking

To predict the binding properties of the newly designed compounds, we employed *in silico* molecular docking to predict similarities and differences in interaction properties between the parent compound CNBDA and the two newly designed compounds, using the molecular modeling program Glide (Schrodinger) with induced-fit docking and binding energy calculation capabilities.^28^ Each molecule was docked into the SHP2 active site, using the crystal structure of the PTP domain (PDB: 4PVG) that was solved in complex with the active site inhibitor 11a-1.^21^ The compounds were also docked into the SHP1 active site using the previously published PTP structure (PDB: 1GWZ) for comparison. Since the compounds are designed to bind to the active site of SHP2, the 20 × 20 × 20 Å^3^ grid box was placed around the point defined by the sulfur atom of the catalytic cysteine (C459 for SHP2 and C455 for SHP1). Prime MM-GBSA calculations were used to predict changes in free energy of binding denoted as Δ*G*. The two-dimensional interaction map was generated from the PDB file of the docked poses, using the MAESTRO 2-D sketcher of Glide.

### Chemistry

For organic synthesis, reagents and solvents were purchased from Sigma-Aldrich (Sigma-Aldrich, TCI, Oakwood, Enamine, and ChemImpex) and were used without addional purifications. ^1^H and ^13^C NMR spectra were recorded on Jeol 400 spectrometers with TMS or residual solvent as standard. Column chromatography was performed on silica gel (100–200 mesh) using a proper eluent. Thin-layer chromatography (TLC) analysis was performed on precoated silica gel 60 F254 plates. Visualization on TLC was achieved by the use of UV light (254 nm). NMR spectra were recorded in chloroform-*d*, methanol-*d*_4_, and DMSO-*d*_6_ at 400 MHz for ^1^H NMR spectra and 100 MHz for ^13^C NMR spectra. Chemical shifts were quoted in parts per million (ppm) referenced to the appropriate solvent peak or 0.0 ppm for tetramethylsilane. The following abbreviations were used to describe peak splitting patterns when appropriate: br = broad, s = singlet, d = doublet, t = triplet, q = quartet, p= pentet (quintet), dd = doublet of doublet, td = triplet of doublet, m = multiplet. Coupling constants (*J*) are reported in hertz (Hz). ^13^C NMR chemical shifts were reported in ppm referenced to the center of a triplet at 77.0 ppm of chloroform-d, 49.0 ppm for methanol-d_4_ and 40.0 ppm center for DMSO-*d*_6_. HRMS spectra were recorded using Quadrupole and Orbitrap LC-MS/MS techniques. The purity of the final products was determined by HPLC, which was performed on an Agilent 1100 Series flexible pump, a vial sampler, and a multicolumn thermostat fitted with a New Phenomenex Hyperclone MOS C8 column 5 μm, 4.6 mm × 150 mm HPLC 00F-4359-E0, a diode array detector, and a 6125B MSD single quadrupole detector (eluent A, 0.1% formic acid in water; eluent B, acetonitrile). The method composition for the HPLC was: 2 min (A: 98.00%, b: 2.00%), 25 min (A: 40.00%, B: 60.00), 27 min (A: 5.0%, B: 95.0%), 29 min (A: 5.0%, B: 95.0%), 30 min (A: 98.0%, B:2.0%). These analyses showed that the final compounds were >95% pure as determined by reverse phase HPLC (λ 250 nm).

#### 3′-Formyl-4′-hydroxy-[1,1′-biphenyl]-4-carbonitrile (**3a**)

Under nitrogen atmosphere, a mixture of 5-Bromosalicylaldehyde (**1**, 804 mg, 4.0 mmol), 4-cyanophenylboronic acid (**2a**, 705, 4.8 mmol), Pd (PPh_3_)_4_ (231 mg, 5 mol %), Na_2_CO_3_ (848 mg, 8.0 mmol) in toluene (10 mL), water (5 mL), and methanol (5 mL) was refluxed for 12 h at 80 °C. After completion of the reaction (by TLC), the mixture was cooled to room temperature, the solvent was evaporated, and the crude residue was quenched with saturated ammonium chloride and extracted with ethyl acetate (50 mL × 3). The extract was washed with water and brine, dried over Na_2_SO_4_, and evaporated. The residue was purified by column chromatography over silica gel (*n*-hexane/ethyl acetate = 6:4) to give **3a** as a white solid (776 mg, 86%). ^1^H NMR (400 MHz, CDCl_3_) δ: 11.10 (s, 1H), 10.00 (s, 1H), 7.76 (m, *J* = 13.3, 7.5 Hz, 3H), 7.68 (t, *J* = 9.6 Hz, 3H), 7.13 (d, *J* = 8.3 Hz, 1H). ^13^C NMR (100 MHz, CDCl_3_) δ: 196.4, 162.0, 143.7, 143.5, 135.5, 132.9, 132.8, 132.2, 127.9, 127.1, 120.8, 118.7.

#### (*E*)-3′-(3-Cyanoprop-1-en-1-yl)-4′-hydroxy-[1,1′-biphenyl]-4-carbonitrile (**4a**)

The Wittig olefination reaction was optimized and used for the synthesis of **4a**. Under nitrogen atmosphere to a round-bottom flask charged with (2-cyanoethyl) triphenylphosphonium bromide (1.3 g, 3.32 mmol, 1.5 equiv), K_2_CO_3_ (453 mg, 3.32 mmol, 1.5 equiv), and H_2_O (45 μL, 2.19 mmol, 1 equiv), the resulting mixture stirred at room temperature for 20 min. To that aldehyde **3a** (490 mg, 2.19 mmol, 1 equiv) was added, and the resulting mixture stirred at room temperature for 16 h. After completion of reaction (by TLC), it was quenched with water and extracted with ethyl acetate (50 mL × 3), organic layer was dried over sodium sulfate and evaporated on rotavapor. The crude residue was purified by column chromatography over silica gel (*n*-hexane/ethyl acetate = 1:1) to give **4a** as a white solid (280 mg, 50%). ^1^H NMR (400 MHz, DMSO-*d*_6_) δ 10.19 (s, 1H), 7.94–7.72 (m, 5H), 7.52 (dd, *J* = 8.4, 1.6 Hz, 1H), 6.98 (d, *J* = 8.5 Hz, 1H), 6.93 (d, *J* = 16.0 Hz, 1H), 6.42 (dt, *J* = 16.0 Hz, 1H), 3.56 (d, *J* = 5.5 Hz, 2H). ^13^C NMR (100 MHz, DMSO-*d*_6_) δ: 156.0, 144.9, 133.1, 129.6, 128.5, 128.0, 127.3, 126.2, 123.8, 119.8, 119.6, 119.2, 116.9, 109.4, 20.8.

#### 3′-(3-Cyanopropyl)-4′-hydroxy-[1,1′-biphenyl]-4-carbonitrile (**5a**)

To a stirred solution of **4a** in ethyl acetate:methanol (1:1, 10 mL) was added Pd–C (5 mol %), and the mixture was stirred under hydrogen atmosphere at room temperature for 12 h. After completion of the reaction by TLC, the mixture was filtered off and solvent was evaporated on rotavapor. The crude residue was purified by column chromatography over silica gel (*n*-hexane/ethyl acetate = 3:1) to give **5a** as a sticky oil (227 mg, 90%). ^1^H NMR (400 MHz, CDCl_3_) δ 7.68 (dd, *J* = 8.2, 2.3 Hz, 2H), 7.64–7.57 (2 H), 7.38–7.32 (m, 2H), 6.84 (dd, *J* = 8.1, 2.3 Hz, 2H), 5.23 (s, *J* = 1.8 Hz, 1H), 2.90–2.76 (t, 2H), 2.38 (t, *J* = 7.1, 2.4 Hz, 2H), 2.04 (m, *J* = 9.0, 4.5 Hz, 2H). ^13^C NMR (100 MHz, CDCl_3_) δ: 154.4, 145.0, 132.6, 132.0, 129.5, 127.1, 126.7, 119.6, 119.0, 116.0, 110.2, 29.3, 25.3, 16.7.

#### 3′-(3-Cyanopropyl)-4′-(nonyloxy)-[1,1′-biphenyl]-4-carbonitrile (**6a**)

Under nitrogen atmosphere, a mixture of **5a** (100 mg, 0.381 mmol) and K_2_CO_3_ (106 mg, 0.762 mmol) in anhydrous acetonitrile 10 mL was stirred at rt for 10 min, and 1-Bromononane (99 mg, 0.476 mmol) was added and the resulting mixture was refluxed for 12 h. After completion by TLC, mixture was cooled down to room temperature and then diluted with water and extracted with ethyl acetate (50 mL × 3). The extract was washed with water and brine, dried over Na_2_SO_4_, and evaporated. The crude oil was purified by column chromatography over silica gel (*n*-hexane/ethyl acetate, 3:1) to give **6a** as a white solid (115 mg, 76%). ^1^H NMR (400 MHz, CDCl_3_) δ 7.69 (d, *J* = 8.3 Hz, 2H), 7.64 (d, *J* = 8.2 Hz, 2H), 7.44 (dd, *J* = 8.4, 2.0 Hz, 1H), 7.38 (d, *J* = 1.8 Hz, 1H), 6.93 (d, *J* = 8.5 Hz, 1H), 4.02 (t, *J* = 6.4 Hz, 2H), 2.85 (t, *J* = 7.3 Hz, 2H), 2.35 (t, *J* = 7.1 Hz, 2H), 2.01 (q, *J* = 7.2 Hz, 2H), 1.89–1.77 (q, 2H), 1.53–1.43 (m, 2H), 1.36–1.24 (m, 10H), 0.89 (t, *J* = 6.5 Hz, 3H). ^13^C NMR (100 MHz, CDCl_3_) δ: 157.6, 145.12, 132.6, 131.0, 129.0, 127.0, 126.7, 119.7, 119.0, 68.1, 31.9, 29.7, 29.5, 29.3, 29.3, 29.2, 26.1, 25.4, 22.7, 16.7, 14.1.

#### 3′-(3-Carboxypropyl)-4′-(nonyloxy)-[1,1′-biphenyl]-4-carboxylic Acid (CNBCA)

To a 1 N NaOH solution (3 mL) was added **6a** (50 mg, 0.128 mmol) in EtOH (3 mL). The resulting mixture was refluxed for 12 h, and when TLC (60% ethyl acetate/hexane) showed that the reaction was completed; the mixture was concentrated under reduced pressure to remove ethanol. The resulting aqueous solution was cooled in an ice bath and made acidic (pH 1) with 1 N HCl to give a precipitate. The aqueous suspension was extracted with ethyl acetate (30 mL x 3). The organic phase was washed with saturated brine (50 mL), dried over sodium sulfate, and filtered, and the filtrate was concentrated under reduced pressure to give a white solid. It was purified by column chromatography over silica gel (*n*-hexane/ethyl acetate, 1:1) to give CNBCA as a white solid (41 mg, 74%). ^1^H NMR (400 MHz, methanol-d_4_) δ 8.05 (d, *J* = 8.0 Hz, 2H), 7.69 (d, *J* = 7.9 Hz, 2H), 7.51 (d, *J* = 8.5 Hz, 1H), 7.47 (s, 1H), 7.01 (d, *J* = 8.5 Hz, 1H), 4.04 (t, *J* = 6.2 Hz, 2H), 2.74 (t, *J* = 7.4 Hz, 2H), 2.32 (t, *J* = 7.4 Hz, 2H), 1.94 (p, *J* = 7.5 Hz, 2H), 1.88–1.79 (m, 2H), 1.53 (dd, *J* = 14.6, 6.9 Hz, 2H), 1.43–1.26 (m, 10H), 0.91 (t, *J* = 6.2 Hz, 3H). ^13^C NMR (100 MHz, methanol-d_4_) δ: 177.5, 169.9, 158.8, 147.0, 133.0, 131.7, 131.3, 129.9, 129.8, 127.4, 127.2, 112.7, 69.0, 34.5, 33.0, 30.9, 30.7, 30.5, 30.5, 30.4, 27.4, 26.4, 23.8, 14.5. HRMS (ESI) [M – H]^−^: *m*/*z* caldt for C_26_H_34_O_5_: 424.5564; found: 424.5564.

#### 2-(3′-Formyl-4′-hydroxy-[1,1′-biphenyl]-4-yl) Acetonitrile (**3b**)

According to the procedure described for the preparation of **3a**, the same procedure was followed for **3b**; under a nitrogen atmosphere, a mixture of 5-bromosalicylaldehyde (**1**, 1g, 0.497 mmol), 4-(4,4,5,5-tetramethyl-1,3,2-dioxaborolan-2-yl) benzeneacetonitrile (**2b**, 1.45g, 0.597 mmol), Pd(PPh_3_)_4_ (287 mg, 5 mol %), Na_2_CO_3_ (1.0 g, 0.994 mmol) in toluene (10 mL), water (5 mL), and methanol (5 mL) was refluxed for 12 h at 80 °C to give **3b** as a yellow solid (950 mg, 82%). ^1^H NMR (400 MHz, CDCl_3_) δ 11.02 (s, *J* = 2.7 Hz, 1H), 9.97 (s, *J* = 2.5 Hz, 1H), 7.75 (dd, *J* = 5.6, 2.4 Hz, 2H), 7.60–7.52 (m, 2H), 7.41 (d, *J* = 6.1 Hz, 2H), 7.08 (dd, *J* = 9.1, 2.6 Hz, 1H), 3.80 (d, *J* = 2.0 Hz, 3H). ^13^C NMR (100 MHz, CDCl_3_) δ: 196.5, 161.2, 139.2, 135.6, 132.2, 131.8, 129.0, 128.6, 127.2, 120.7, 118.3, 117.7, 23.3.

#### (*E*)-4-(4′-(Cyanomethyl)-4-hydroxy-[1,1′-biphenyl]-3-yl) But-3-enenitrile (**4b**)

According to the procedure described for the preparation of **4a**, the same procedure was followed for **4b**; under nitrogen atmosphere to a round-bottom flask charged with (2-cyanoethyl) triphenylphosphonium bromide (521 mg, 1.31 mmol, 1.3 equiv), K_2_CO_3_ (210 mg, 1.51 mmol, 1.3 equiv), and H_2_O (19 μL, 1.0 mmol, 1 equiv), resulting mixture stirred at room temperature for 20 min. To that aldehyde **3b** (240 mg, 1.0 mmol, 1 equiv) was added, and the resulting mixture was stirred at room temperature for 16 h to obtain **4b** as a light yellow solid (165 mg, 59%). ^1^H NMR (400 MHz, CDCl_3_) δ: 7.52 (d, *J* = 6.9 Hz, 3H), 7.38–7.30 (m, 3H), 6.98 (d, *J* = 16.1 Hz, 1H), 6.87 (d, *J* = 8.3 Hz, 1H), 6.21 (dt, *J* = 15.7, 5.7 Hz, 1H), 6.07 (s, *J* = 12.2 Hz, 1H), 3.78 (s, 2H), 3.32 (d, *J* = 5.7 Hz, 2H). ^13^C NMR (100 MHz, CDCl_3_) δ: 153.2, 140.3, 132.8, 129.6, 128.3, 128.2, 127.7, 127.3, 126.4, 123.3, 122.7, 118.4, 118.0, 117.6, 116.5, 23.2, 21.2.

#### 4-(4′-(Cyanomethyl)-4-hydroxy-[1,1′-biphenyl]-3-yl) Butanenitrile (**5b**)

To a stirred solution of **4b** (90 mg, 0.328 mmol) in ethyl acetate:methanol (1:1, 12 mL) was added Pd–C (5 mol %), and the mixture was stirred under hydrogen atmosphere at room temperature for 12 h. After completion of the reaction by TLC, the mixture was filtered off and solvent was evaporated on rotavapor. The crude residue was purified by column chromatography over silica gel (*n*-hexane/ethyl acetate = 3:1) to give **5b** as a light yellow solid (78 mg, 86%). ^1^H NMR (400 MHz, CDCl_3_) δ 7.54 (d, *J* = 8.1 Hz, 2H), 7.40–7.29 (m, 4H), 6.83 (d, *J* = 8.1 Hz, 1H), 5.33 (s, *J* = 5.9 Hz, 1H), 3.79 (s, 2H), 2.85 (t, *J* = 7.4 Hz, 3H), 2.39 (t, *J* = 7.1 Hz, 2H), 2.05 (p, *J* = 7.3 Hz, 2H). ^13^C NMR (100 MHz, CDCl_3_) δ: 153.7, 140.5, 132.9, 129.2, 128.3, 128.1, 127.3, 126.7, 126.4, 119.8, 117.9, 115.8, 29.3, 25.3, 23.3, 16.6.

#### 4-(4′-(Cyanomethyl)-4-(nonyloxy)-[1,1′-biphenyl]-3-yl) Butanenitrile (**6b**)

According to the procedure described for the preparation of **6a**, the same procedure was followed for **6b**; under nitrogen atmosphere, a mixture of **5b** (35 mg, 0.126 mmol) and K_2_CO_3_ (22 mg, 0.158 mmol) in anhydrous acetonitrile 5 mL was stirred at room temperature for 10 min, to that 1-bromononane (33 mg, 0.158 mmol) was added, and the resulting mixture was refluxed for 12 h to furnish **6b** as a yellow liquid (42 mg, 80%). ^1^H NMR (400 MHz, CDCl_3_) δ 7.55 (d, *J* = 7.7 Hz, 2H), 7.39 (m, *J* = 16.8, 8.8 Hz, 4H), 6.91 (d, *J* = 8.4 Hz, 1H), 4.01 (t, *J* = 6.4 Hz, 2H), 3.78 (s, 2H), 2.84 (t, *J* = 7.2 Hz, 2H), 2.35 (t, *J* = 7.1 Hz, 2H), 2.08–1.94 (q, 2H), 1.89–1.77 (q, 2H)1.47 (m, *J* = 14.1, 6.9 Hz, 2H), 1.29 (m, 10H), 0.89 (t, *J* = 6.0 Hz, 3H). ^13^C NMR (100 MHz, CDCl_3_) δ: 156.8, 140.6, 132.3, 128.9, 128.7, 128.3, 128.1, 127.3, 126.3, 119.8, 117.9, 111.5, 68.0, 31.9, 29.7, 29.5, 29.3, 29.3, 26.2, 25.5, 23.3, 22.7, 16.7.

#### 4-(4′-(Carboxymethyl)-4-(nonyloxy)-[1,1′-biphenyl]-3-yl) Butanoic Acid (CNBBA)

To a 1 N NaOH solution (3 mL) was added **6b** (27 mg, 0.067 mmol) in EtOH (pH 3 0) (3 mL). The resulting mixture was refluxed for 12 h, and when TLC (60% ethyl acetate/hexane) showed that the reaction was completed, the mixture was concentrated under reduced pressure to remove ethanol. The resulting aqueous solution was cooled in an ice bath and made acidic (pH 1) with 1 N HCl to give a precipitate. The aqueous suspension was extracted with an ethyl acetate (25 mL × 3). The organic phase was washed with saturated brine (50 mL), dried over sodium sulfate, and filtered, and the filtrate was concentrated under reduced pressure to give a white solid. It was purified by column chromatography over silica gel (*n*-hexane/ethyl acetate, 1:1) to give CNBBA as a light yellow solid (24 mg, 75%). ^1^H NMR (400 MHz, CDCl_3_) δ 7.49 (d, *J* = 7.6 Hz, 2H), 7.34 (m, *J* = 8.8 Hz, 4H), 6.87 (d, *J* = 8.2 Hz, 1H), 3.98 (t, *J* = 6.3 Hz, 2H), 3.68 (s, 2H), 2.73 (t, *J* = 7.3 Hz, 2H), 2.39 (t, *J* = 7.1 Hz, 2H), 2.08–1.91 (q, 2H), 1.88–1.74 (q, 2H), 1.53–1.42 (m, 2H), 1.30 (m, *J* = 20.7 Hz, 10H), 0.89 (t, *J* = 6.1 Hz, 3H). ^13^C NMR (100 MHz, CDCl_3_) δ: 179.9, 177.7, 156.6, 140.3, 132.9, 131.6, 129.9, 129.7, 129.0, 127.0, 125.7, 111.3, 68.0, 40.8, 33.3, 31.9, 29.6, 29.4, 29.3, 26.2, 24.6, 22.7, 14.0. HRMS (ESI) [M – H]^−^: *m*/*z* caldt for C_27_H_36_O_5_: 438.5864; found: 438.5864.

### PTPase Assay

We used the PTPase assay described previously^30,31^ to evaluate the effect of the newly synthesized compounds on the enzymatic activity of SHP2, SHP1, and PTP1B. The PTP domains of the indicated proteins were prepared as reported by us recently.^32^ The artificial PTP substrate DiFMUP (6,8-Difluoro-4-Methylumbelliferyl Phosphate) was purchased from Invitrogen. The purified proteins were dialyzed into a phosphatase buffer containing 10 mM Tris-HCl (pH 7 7 2), 100 mM NaCl, 1.0 mM EDTA, 1.0 mM dithiothreitol (DTT), and 0.01% Tween-20. Briefly, the PTPase reactions were performed in 100 μL volume containing 1 nM enzyme, inhibitors in serial dilutions (12 nM to 100 μM), and DiFMUP to a final concentration of 20 μM for SHP2, 35 μM for SHP1, and 10 μM for PTP1B. Differences in DiFMUP concentrations reflect the reported Km values for each PTP.^37^ The mixtures were incubated at 37 °C for 20 min and transferred to 96 well plates, and the fluorescence intensity was measured by a Synergy 4 plate reader at the excitation and emission wavelengths of 360 and 460 nm, respectively. Graphpad Prism software was used to calculate the IC_50_ values.

For determining the inhibitory effect of CNBCA on FL-SHP2, a FLAG-tagged protein ectopically expressed in the EGFR overexpressing MDA-MB468 cells^8,15^ was used as an enzyme. First, the protein was purified by immunoprecipitation with anti-FLAG antibody from total protein extracts (TPEs). Next, SHP2 bound to the beads was equally divided into 12 wells, mixed with varying concentration CNBCA prepared by serial dilution (95 nM – 100 μM) in the PTPase buffer described above, and incubated at room temperature for 5 min to allow binding. The PTPase reaction was started by adding DiFMUP to a final concentration of 20 μM in approximately 120 μL of total volume, which includes the beads. After 20 min of incubation at 37 °C, 100 μL of the supernatant from each sample was transferred to 96-well plates, and fluorescence intensity measure as above, using the Synergy 4 plate reader. The resulting data was then analyzed with the Graphpad Prism software to draw the graph and determine the IC_50_ value.

To confirm that CNBCA competitively inhibits SHP2, we conducted enzyme kinetic studies using the SHP2 enzyme at 1.0 nM, DiFMUP in serial dilutions in 96-well plates, ranging from 1 to 1024 μM, and in the absence and presence of two different concentrations of CNBCA (1.0 and 2.0 μM). In these reactions, the enzyme was added last, and the fluorescence was read for 3 min at 10 s interval. Changes in fluorescence intensity were used to calculate the *K*_m_ of the SHP2 enzyme for DiFMUP in the presence and absence of CNBCA. The Graphpad Prism was used to prepare the Michaelis–Menten and Lineweaver–Burk plots and to determine the *Km* values.

### Cellular Thermal Shift Assay (CETSA)

For the CETSA study, total protein extracts (TPE) prepared from two breast cancer cells, the triple-negative MDA-MB468 and the HER2-positive JIMT-1 cells, were used. Briefly, TPE from the two cell lines were cleared by centrifugation, mixed with CNBCA at 100 μM concentration (the maximum amount used in the IC_50_ studies), and incubated for 10 min at room temperature to allow binding. The mixture was then divided into 100 μL aliquots, treated with heat for 10 min at different temperatures, ranging from 37 to 64 °C that differ by 3 °C intervals, cooled on ice for 10 min, centrifuged at 20,000g for 20 min to remove precipitated proteins, and the supernatants analyzed by immunoblotting (IB) for SHP2 and the nontarget PTP, PTP1B. Aliquots that were mixed with CNBCA, but not treated with heat were used as positive controls for comparison.

### Cells and Reagents

Both the HER2-positive BT474 and the triple-negative MDA-MB468 breast cancer cell lines were purchased from the American Tissue Culture Collection (ATCC). While the BT474 cells were grown in RPMI, the MDA-MB468 cells were grown in Dulbecco’s modified Eagle’s medium (DMEM), both supplemented with 10% fetal bovine serum. The artificial substrate DiFMUP was purchased from Invitrogen, while glutathione-sepharose beads used for purification of GST fusion PTP domains were purchased from GE Healthcare. The antibodies used in this study, the antiphospho-ERK1/2, anti-phospho-Akt, and anti-panAkt antibodies, were from Cell Signaling biotechnology, the anti panERK2 antibody was from BD Biosciences, and the anti-β-actin antibody was from Sigma-Aldrich.

### Colony Formation Assay

For colony formation studies, we used the soft agar assay, which keeps cells anchorage independently, allowing determination on the transformation phenotype of cancer cells, including breast cancer cells.^11,35^ For this assay, we first prepared a 5% stock suspension in PBS by boiling it in a microwave. Just before use, the solidified agar suspension was boiled and allowed to cool to approximately 45 °C in a water bath and added to cell suspensions in 2 mL of growth medium at a final concentration of 0.3%. The mixture was immediately poured into 6 cm culture plates, allowed to solidify for about 5 min at room temperature, and incubated in a cell culture incubator with 50% CO_2_ at 37 °C for approximately 10 days. Vehicle or CNBDA at 500 nM was added to the cells before mixing with the agar, which was maintained for the 10 day incubation time. Colony formation was visualized by imaging under an Olympus IX71 microscope equipped with a DP80 camera.

### Mammosphere Formation Assay

To further evaluate the anti-breast cancer cell effect of CNBCA, we treated cells seeded in a suspension culture known as the mammosphere assay. In this assay, cells with cancer stem cell properties can grow and form sphere-like cellular aggregates known as mammospheres.^7,38^ For this assay, approximately 10^6^ cells were seeded in serum-free DMEM containing 1 μg/mL hydrocortisone, 10 μg/mL insulin, 10 ng/mL EGF, 10 ng/mL FGF, 5 ng/mL heparin, and B27 (Invitrogen) in 6 cm ultralow adherence culture plates and then treated with vehicle or CNBCA at 500 nM final concentration for approximately 10 days. After these days, mammospheres were collected in smaller volume of media using centrifugation, and imaged using the 10× objective in an Olympus IX71 microscope equipped with DP30 camera. Representative images for each experimental group are used to show effectiveness of the anti-SHP2 compound CNBCA.

## Supporting Information Available

Docking data to the SHP1 active site, ^1^H and ^13^C NMR spectra of intermediates and final products, and HPLC analysis data (PDF)
