# Supplementary material for: Targeting SHP2 with an Active Site Inhibitor Blocks Signaling and Breast Cancer Cell Phenotypes
Source: ACS Bio Med Chem Au. 2023 Jul 14;3(5):418–28. doi: 10.1021/acsbiomedchemau.3c00024 (PMC10591299; doi:10.1021/acsbiomedchemau.3c00024)
Supplement: Supplementary file 1 — bg3c00024_si_001.pdf [file bg3c00024_si_001.pdf]

Supporting Information

Targeting SHP2 with an active site inhibitor blocks signaling and breast cancer cell phenotypes

Dhanaji M. Lade[1] and Yehenew M. Agazie*[1,2]
Department of Biochemistry and Molecular Medicine[1], and WVU Cancer Institute[2], Health Sciences Center, West Virginia University, Morgantown, WV 26506

Contents

Molecular docking to the SHP1 active site	S1
^{1}H and ^{13}C NMR spectrum	S2 - S11
HPLC spectrum	S12 - 15

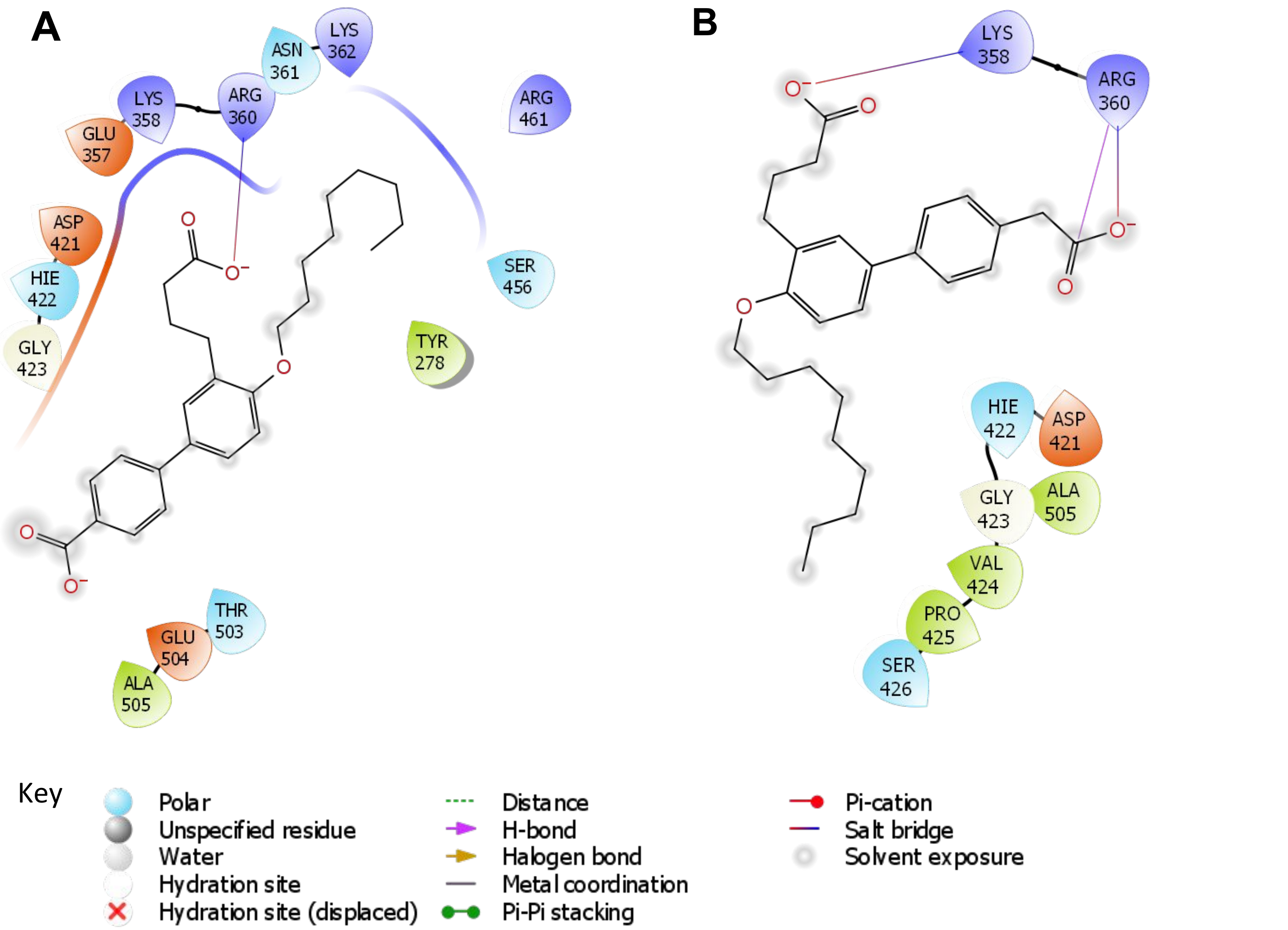

Supporting Figure 1: Molecular docking to the SHP1 active using the PTP domain structure (ID: PDB:1GWZ). A. Interaction map of CNBCA to the SHP1 active site. B. Interaction map of CNBBA to the SHP1 active site.

1) *3'-formyl-4'-hydroxy-[1,1'-biphenyl]-4-carbonitrile* ***(3a)***

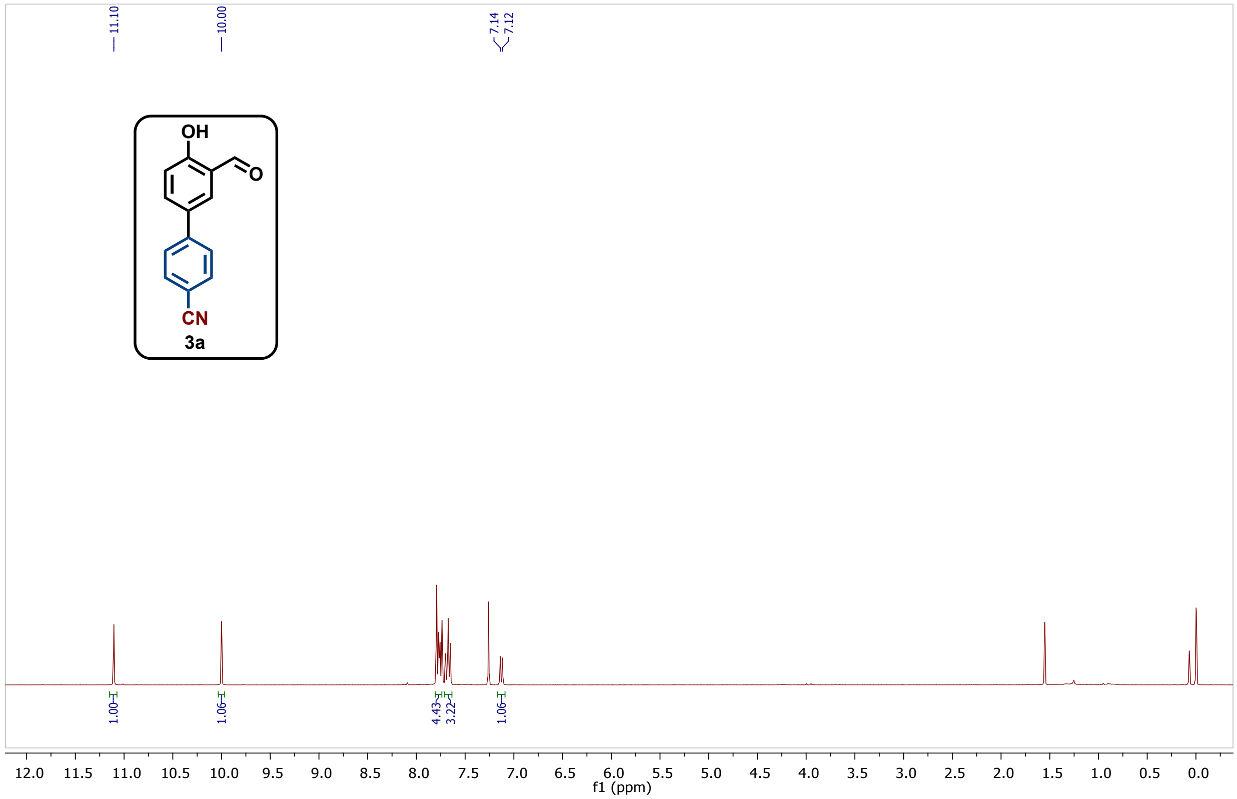

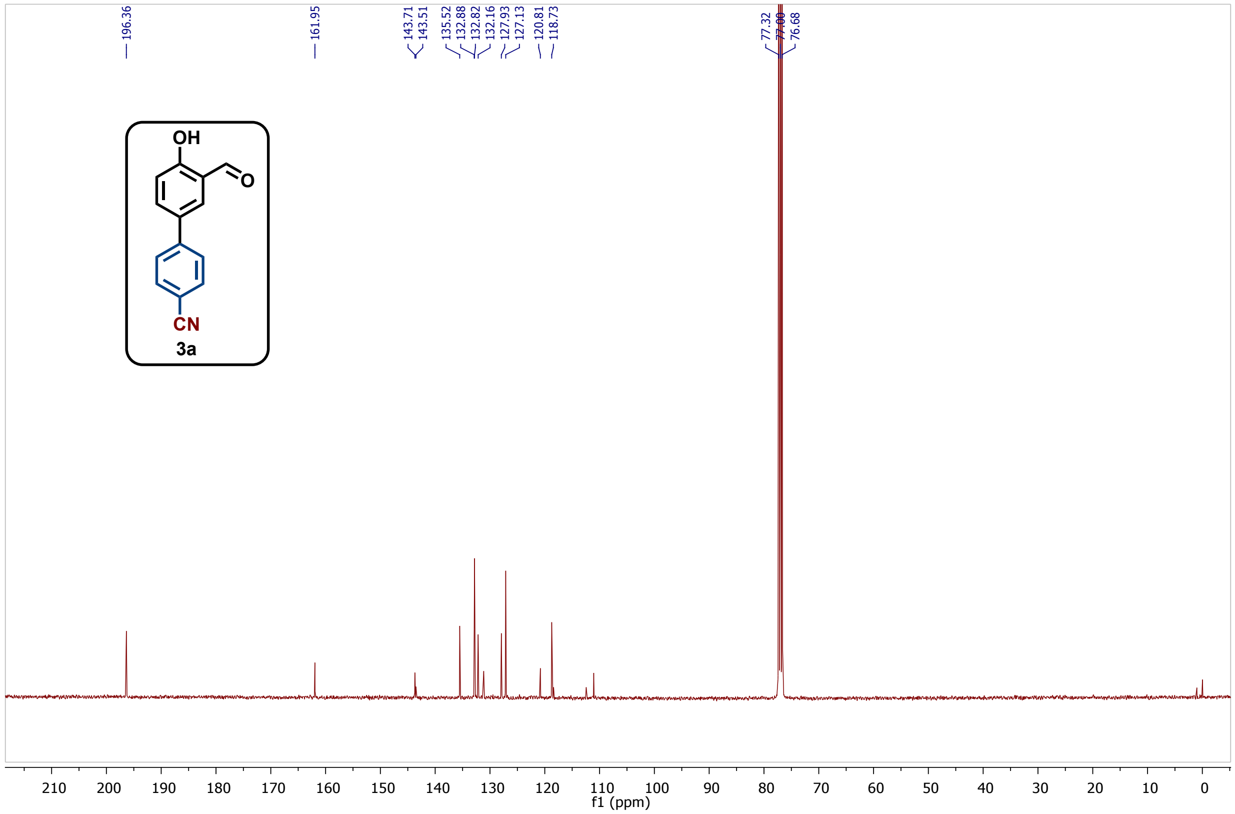

2) *(E)-3'-(3-cyanoprop-1-en-1-yl)-4'-hydroxy-[1,1'-biphenyl]-4-carbonitrile* ***(4a)***

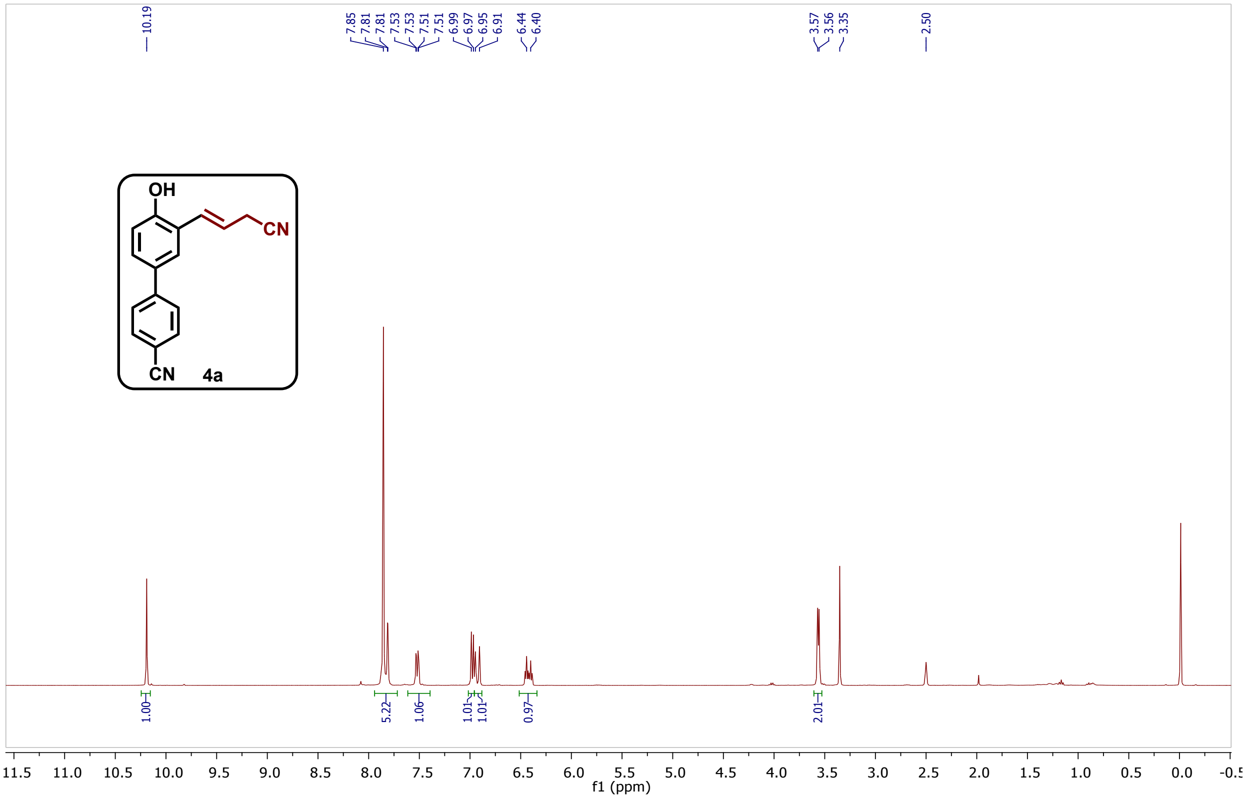

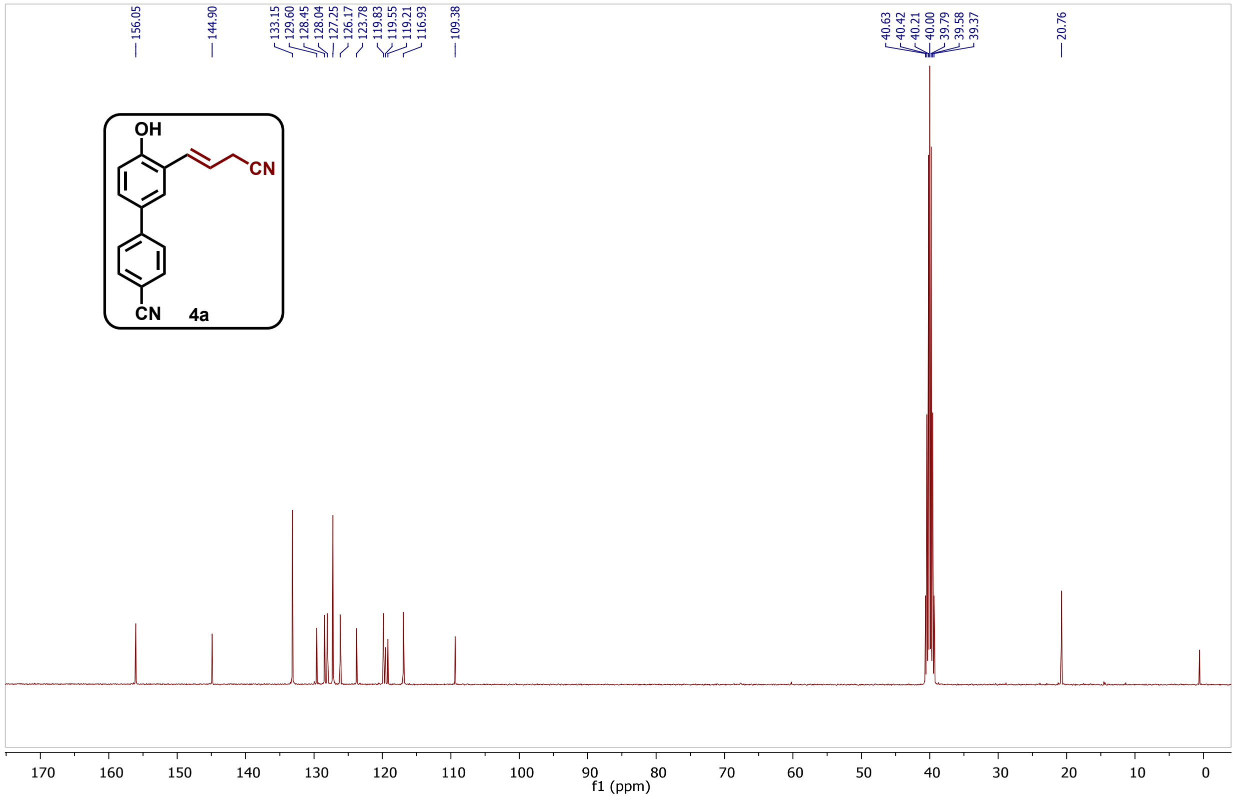

3) *3'-(3-cyanopropyl)-4'-hydroxy-[1,1'-biphenyl]-4-carbonitrile* ***(5a)***

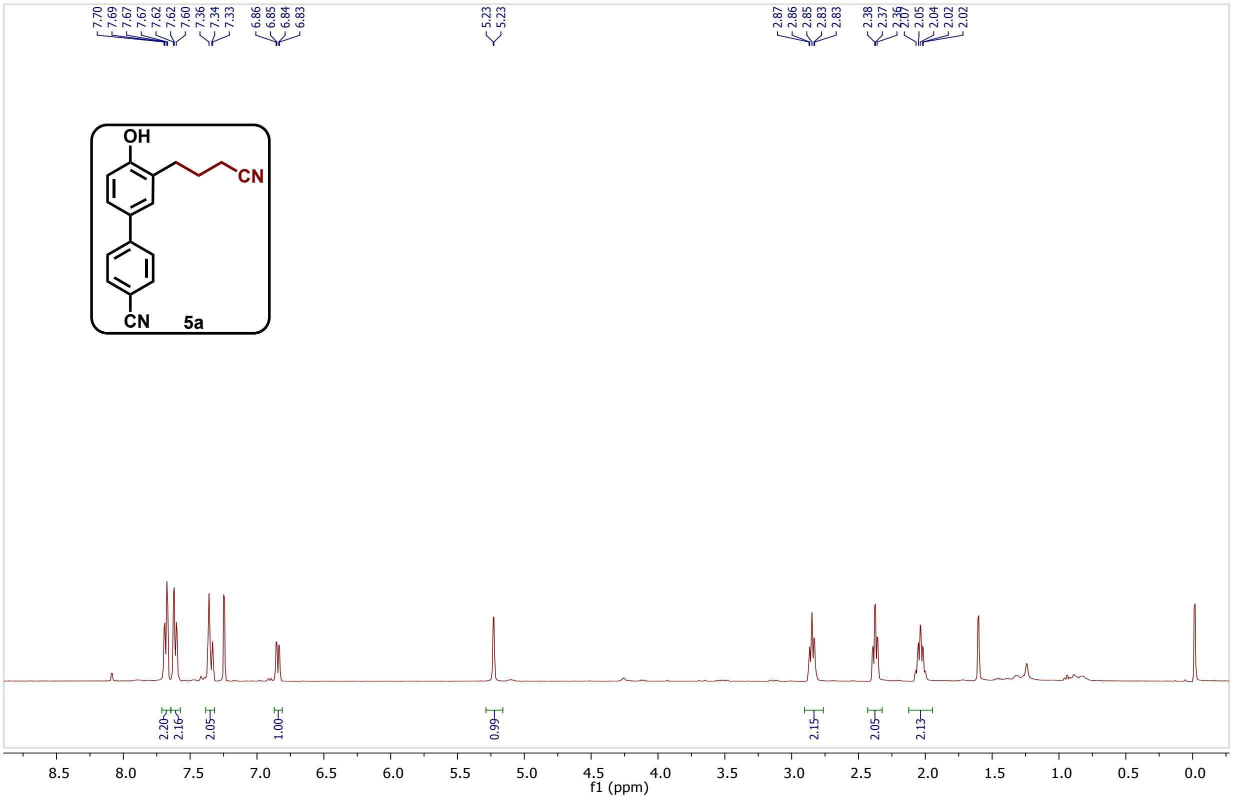

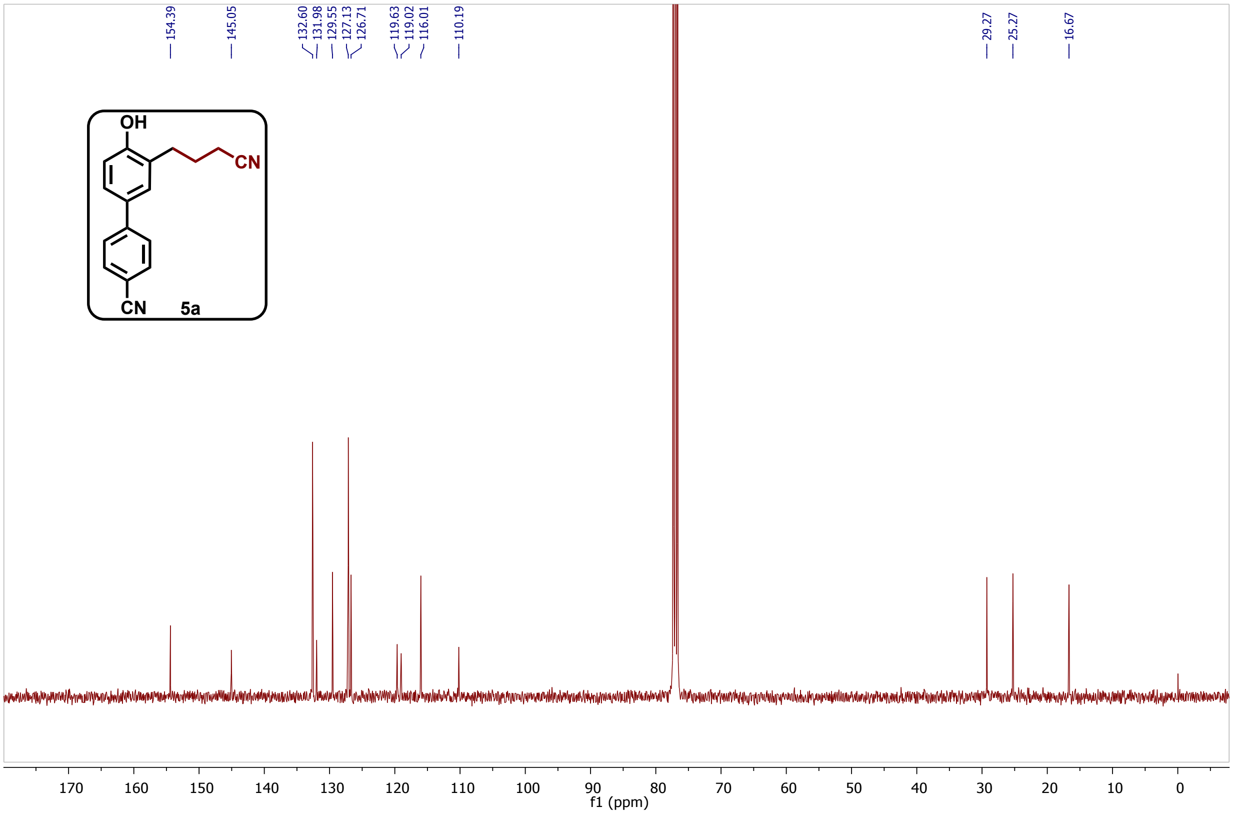

4) *3'-(3-cyanopropyl)-4'-(nonyloxy)-[1,1'-biphenyl]-4-carbonitrile* ***(6a)***

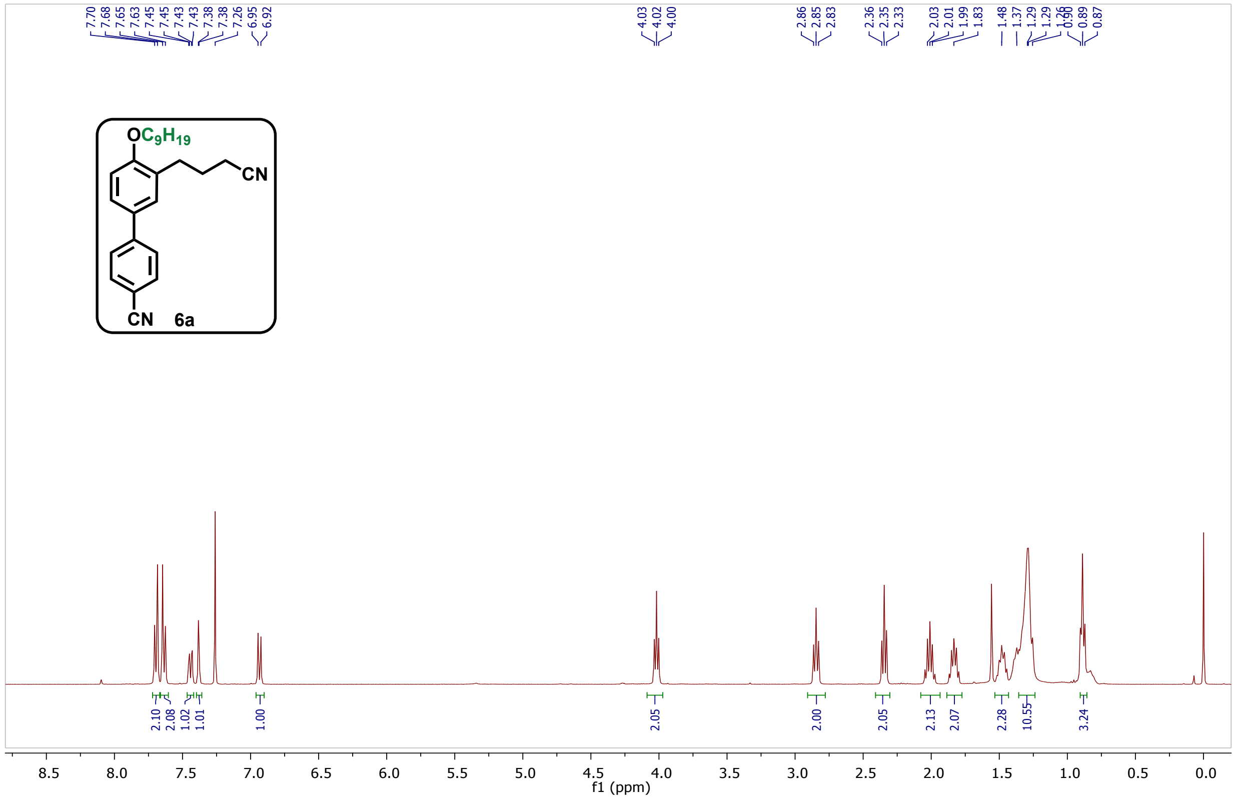

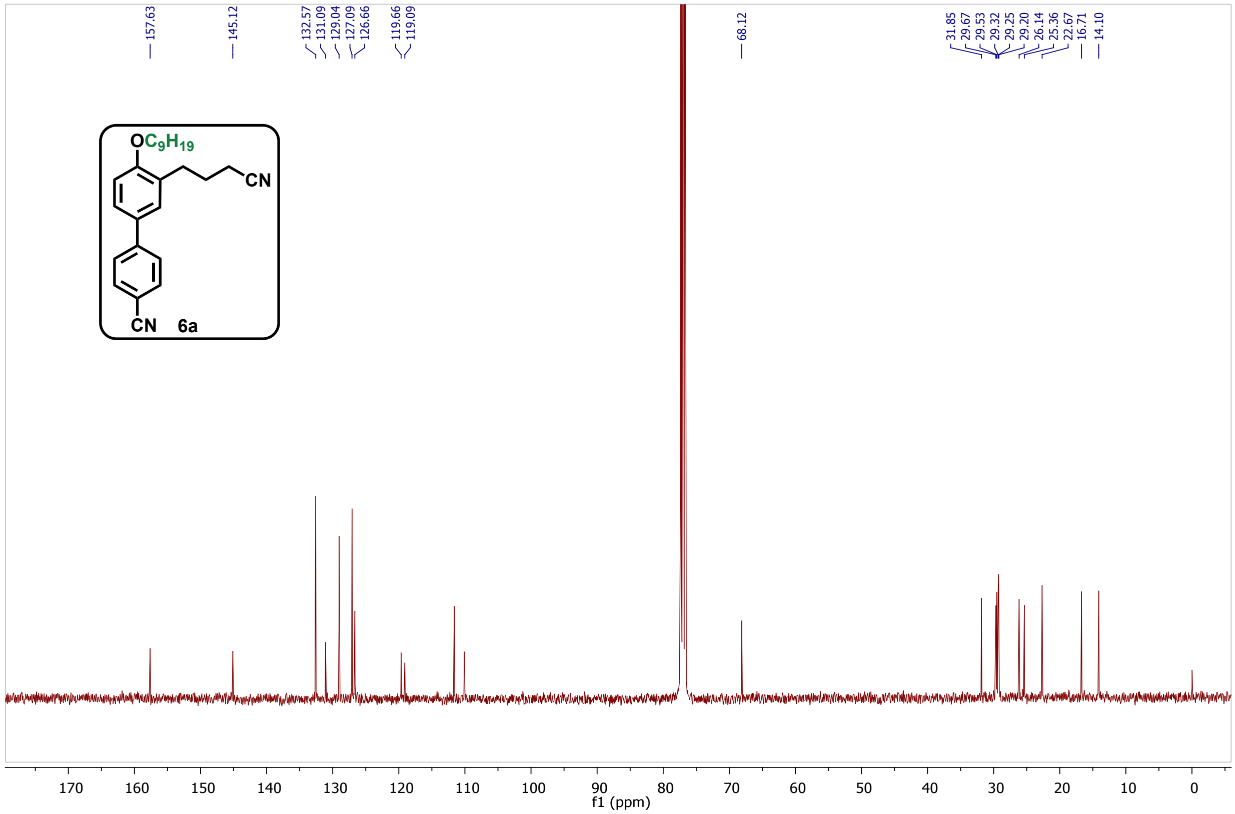

5) *3'-(3-carboxypropyl)-4'-(nonyloxy)-[1,1'-biphenyl]-4-carboxylic acid* (***CNBCA***)

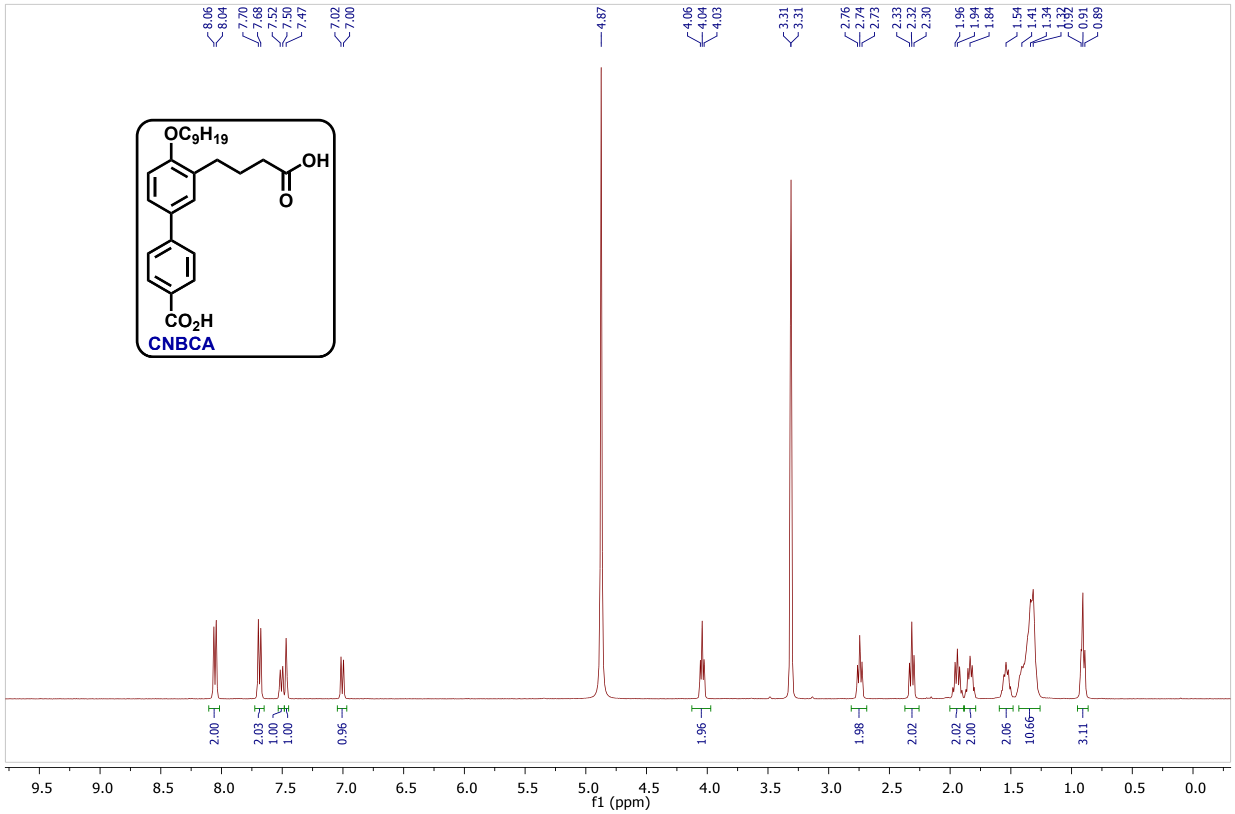

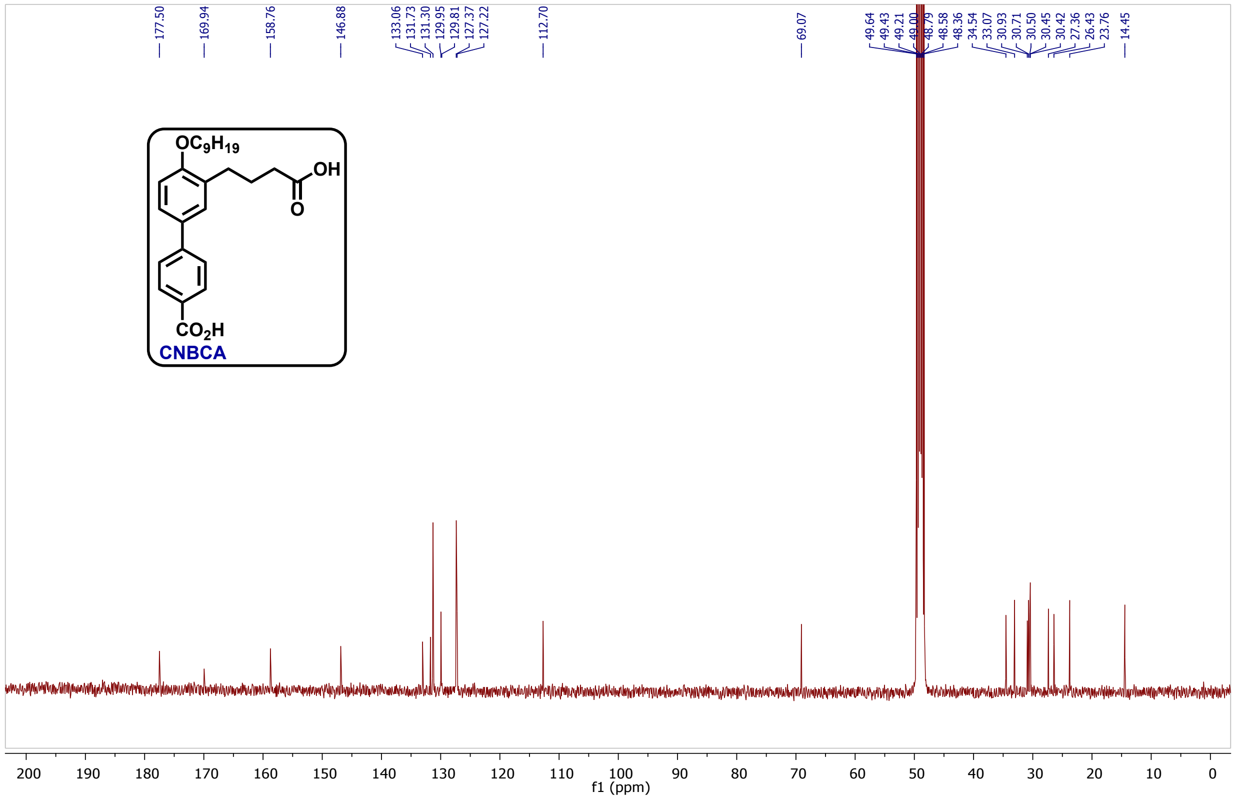

6) *2-(3'-formyl-4'-hydroxy-[1,1'-biphenyl]-4-yl)acetonitrile* ***(3b)***

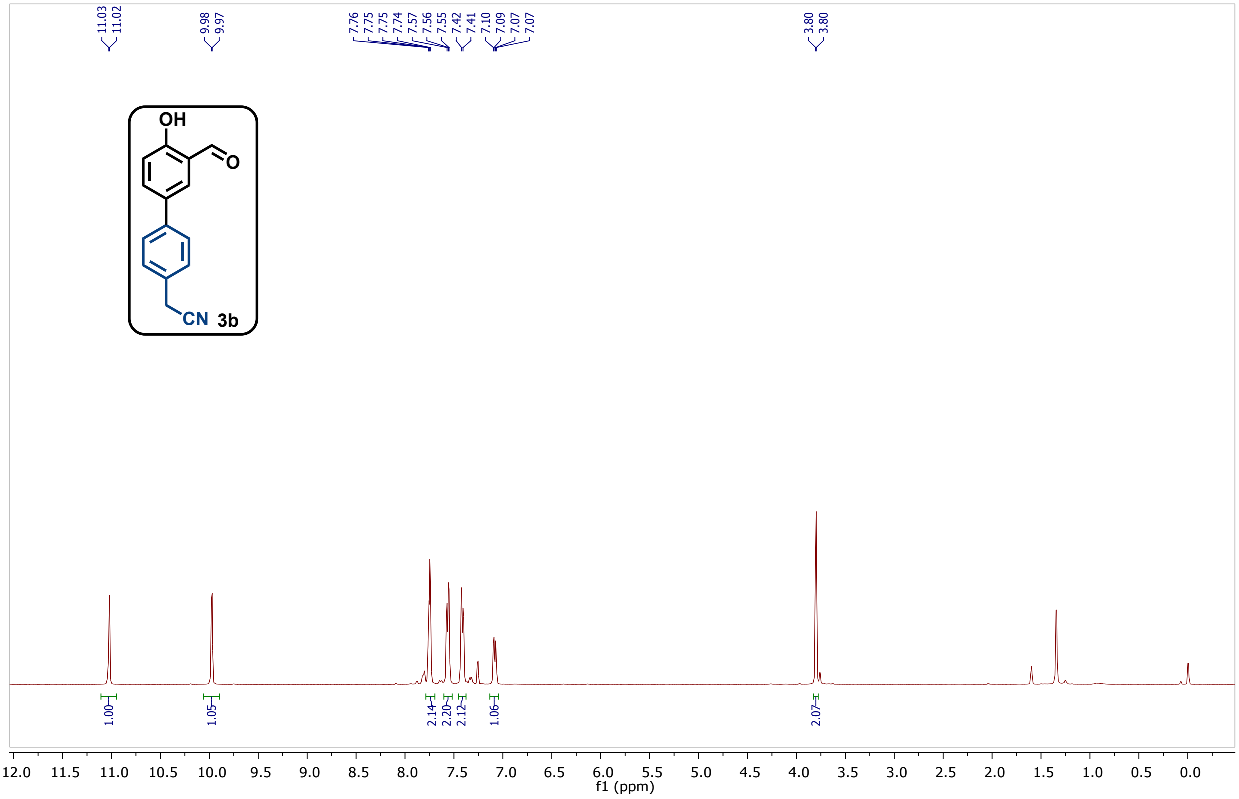

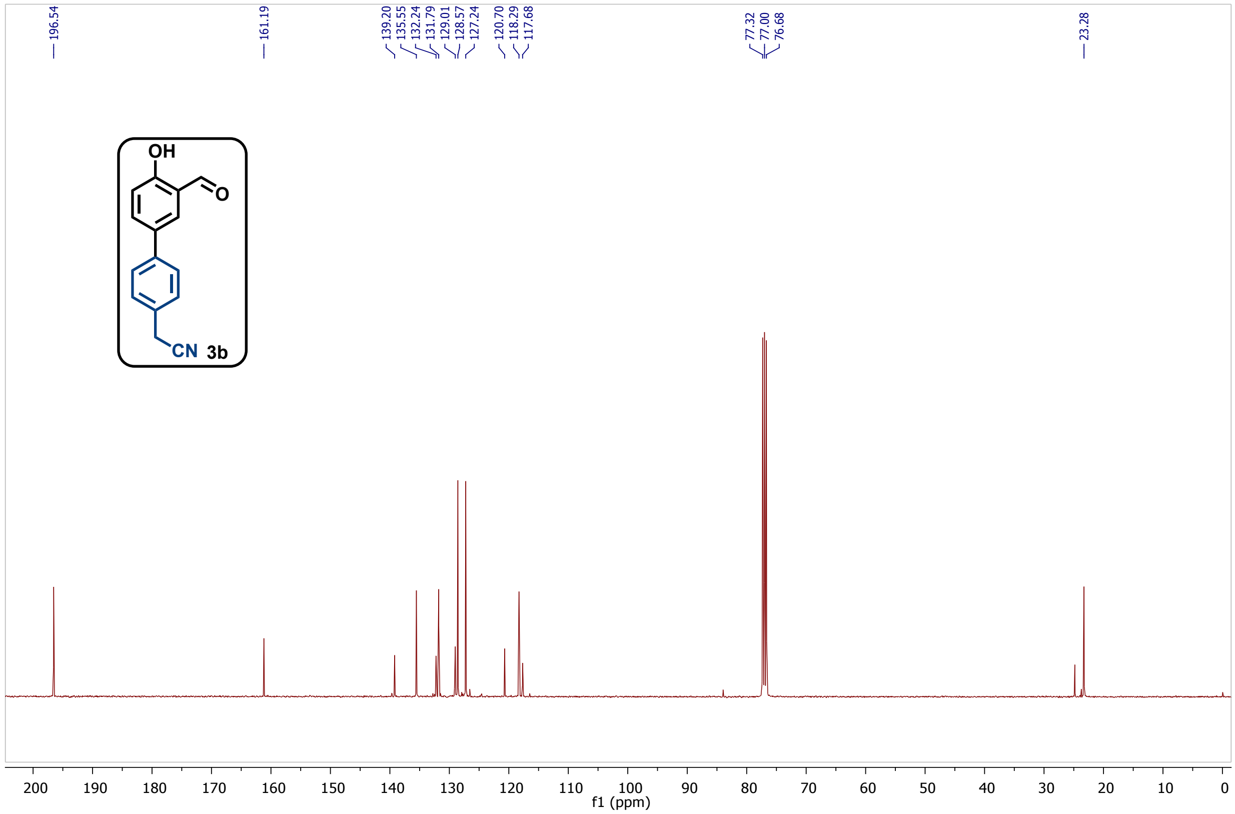

7) *(E)-4-(4'-(cyanomethyl)-4-hydroxy-[1,1'-biphenyl]-3-yl)but-3-enenitrile (**4b**)*

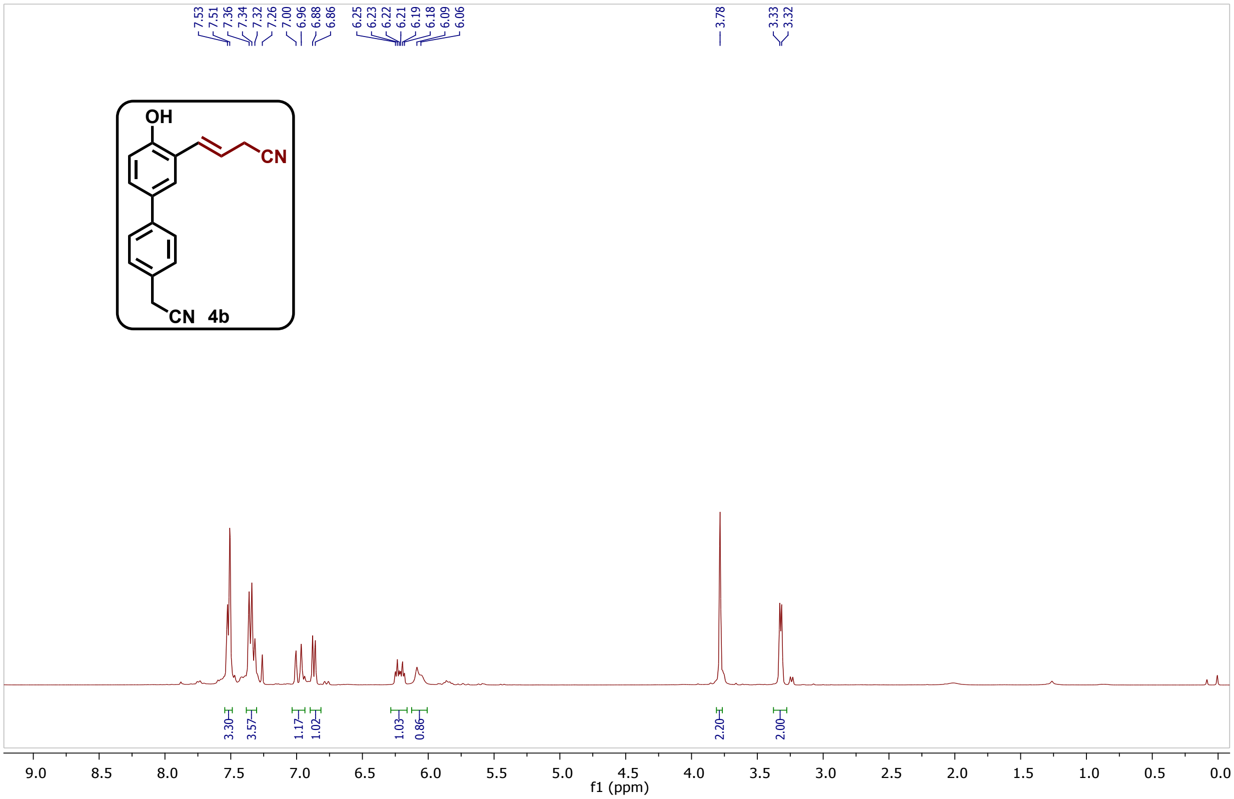

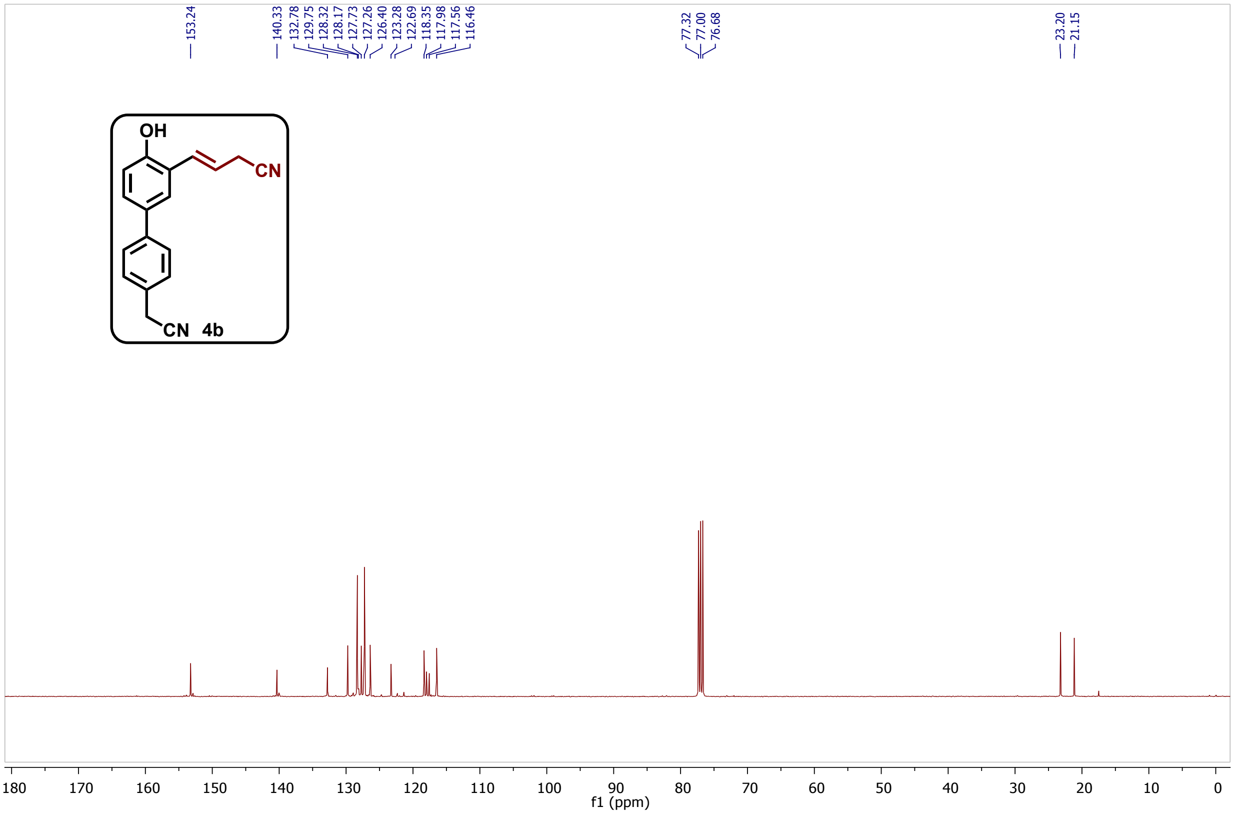

8) *4-(4'-(cyanomethyl)-4-hydroxy-[1,1'-biphenyl]-3-yl)butanenitrile **(5b)***

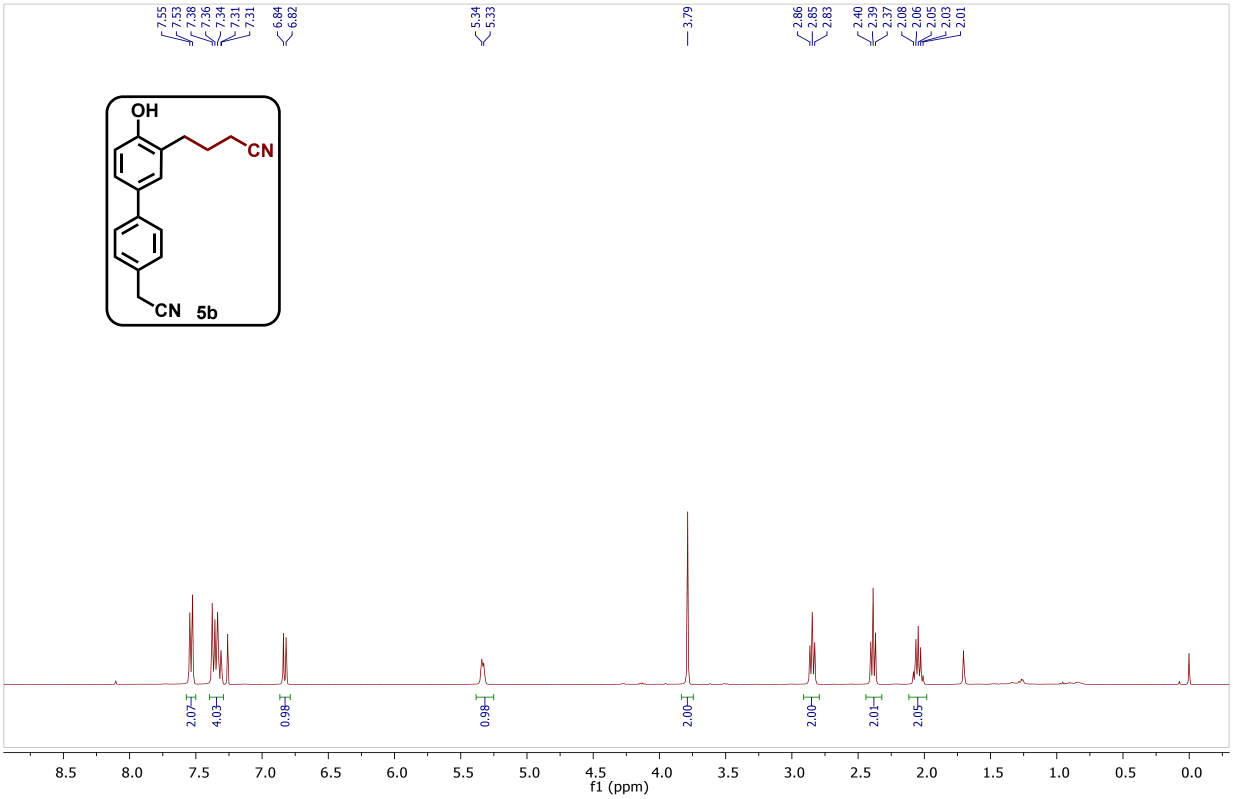

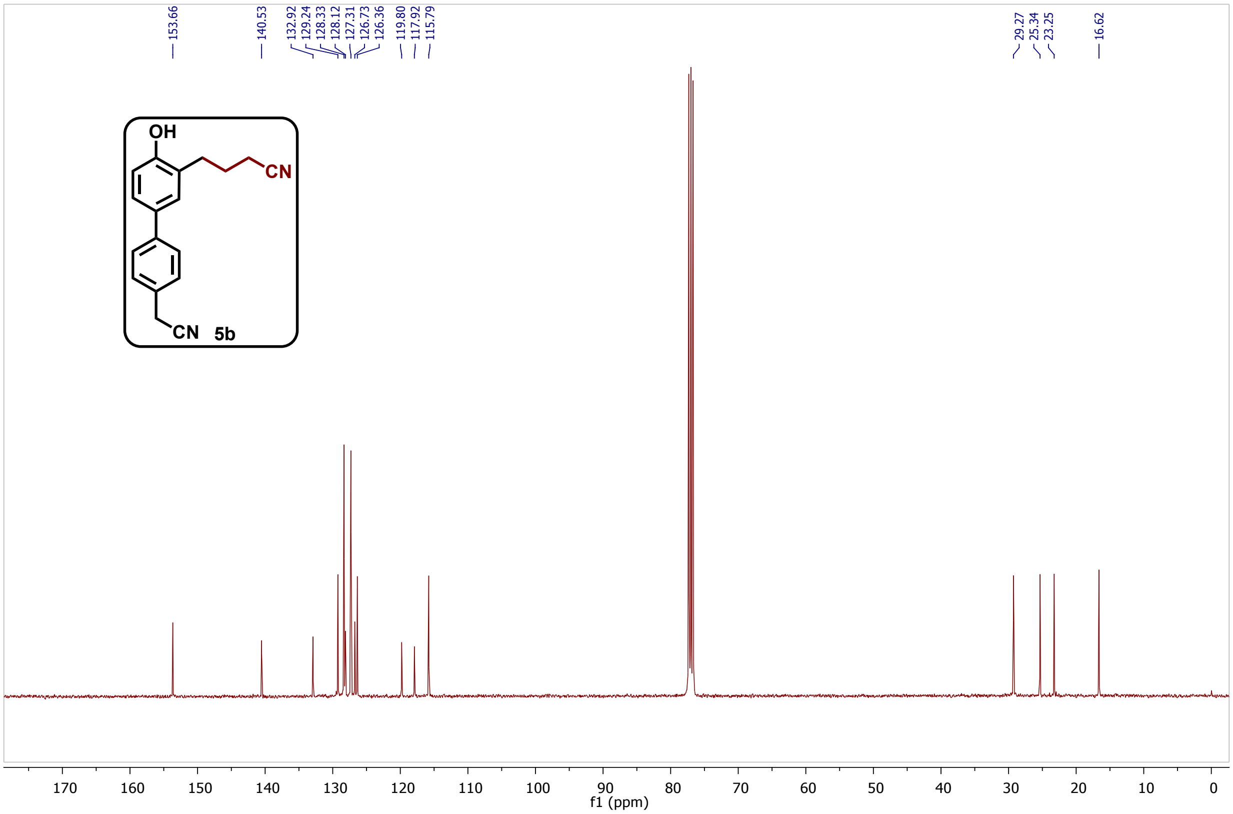

9) *4-(4'-(cyanomethyl)-4-(nonyloxy)-[1,1'-biphenyl]-3-yl)butanenitrile* ***(6b)***

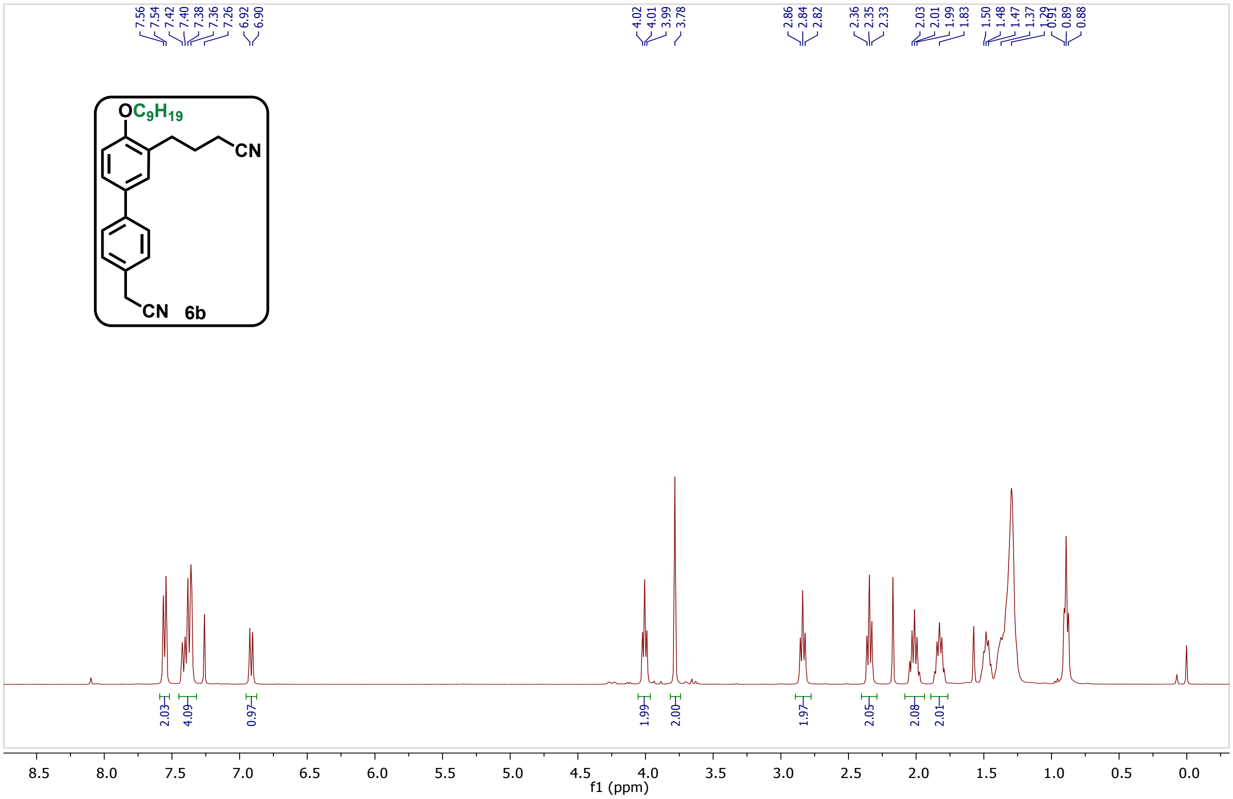

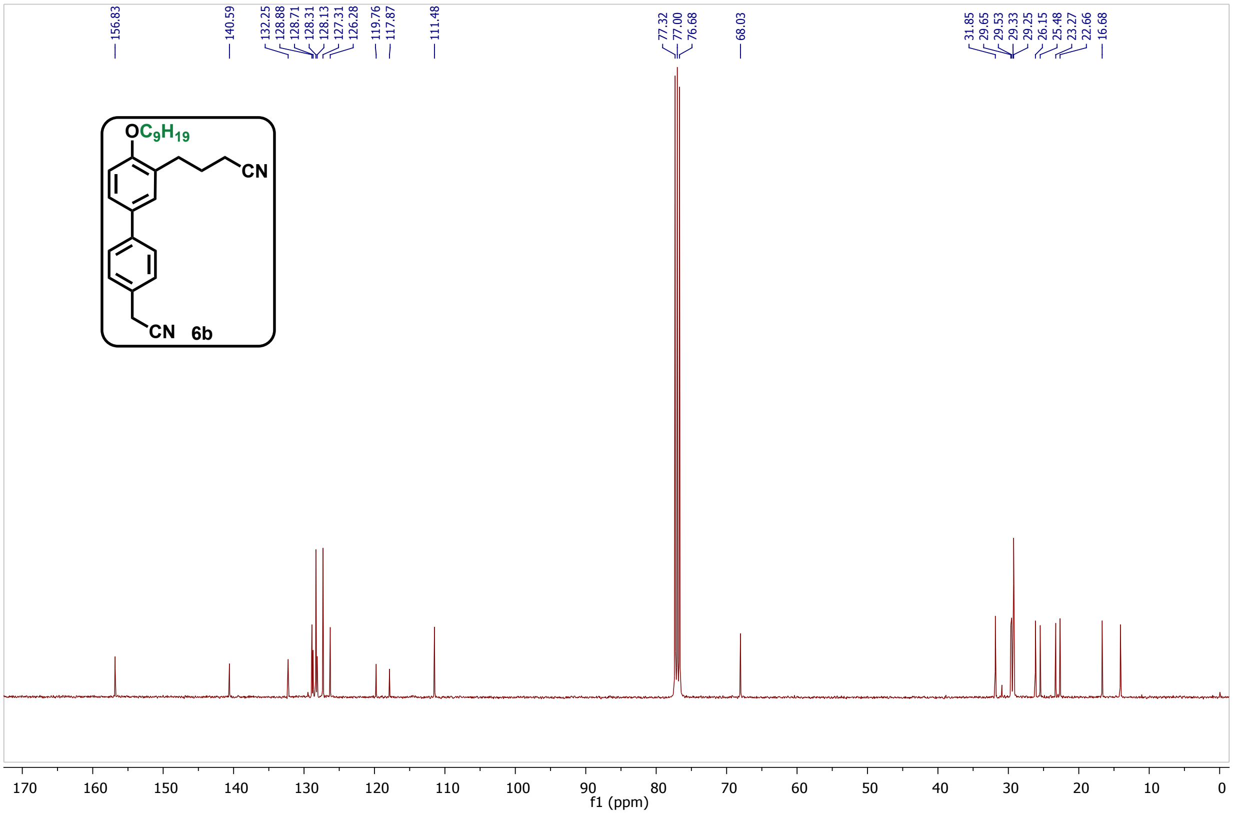

10) *4-(4'-(carboxymethyl)-4-(nonyloxy)-[1,1'-biphenyl]-3-yl)butanoic acid (**CNBBA**)*

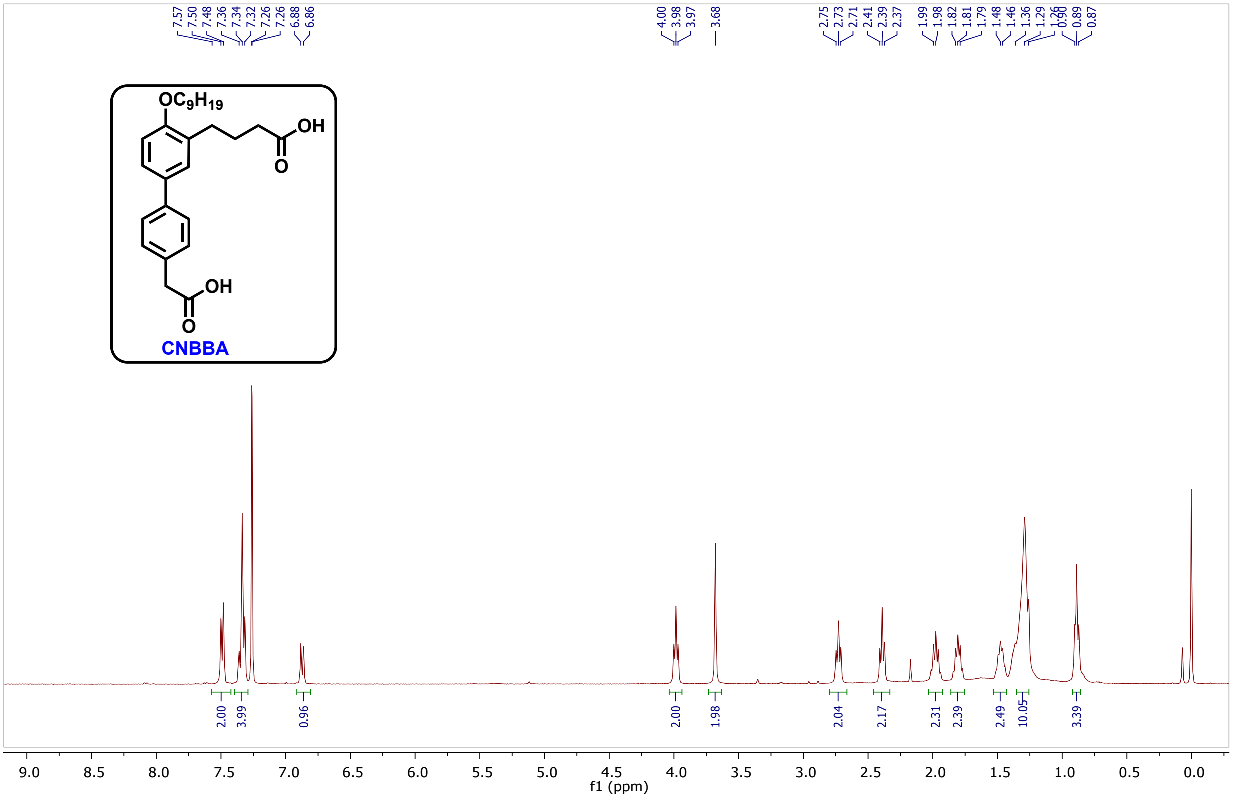

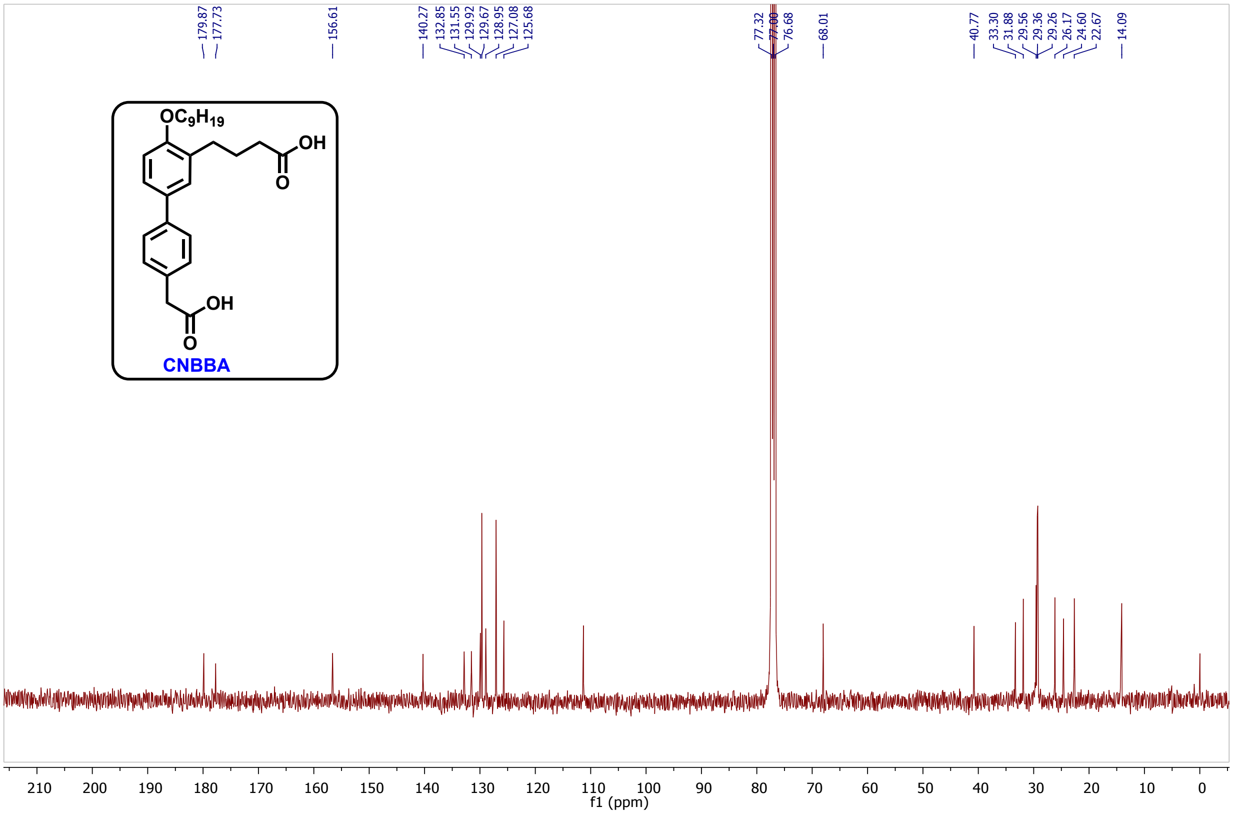

HPLC_CNBCA

```
=====================================================================
Acq. Operator   : Dhanaji
Acq. Instrument : Instrument 1                    Location : Vial 1
Injection Date  : 3/2/2023 11:35:29 AM
                                                Inj Volume : 50.000 µl
Acq. Method     : C:\CHEM32\1\METHODS\NL1_ANALYSIS.M
Last changed    : 3/2/2023 11:33:06 AM by Dhanaji
                  (modified after loading)
Analysis Method : C:\CHEM32\1\METHODS\SRIDHAR-MTI201-AHX-DOTA-2-60-20MIN.M
Last changed    : 3/7/2023 3:34:17 PM by Dhanaji
                  (modified after loading)
Sample Info     : CNBCA_Pure
```

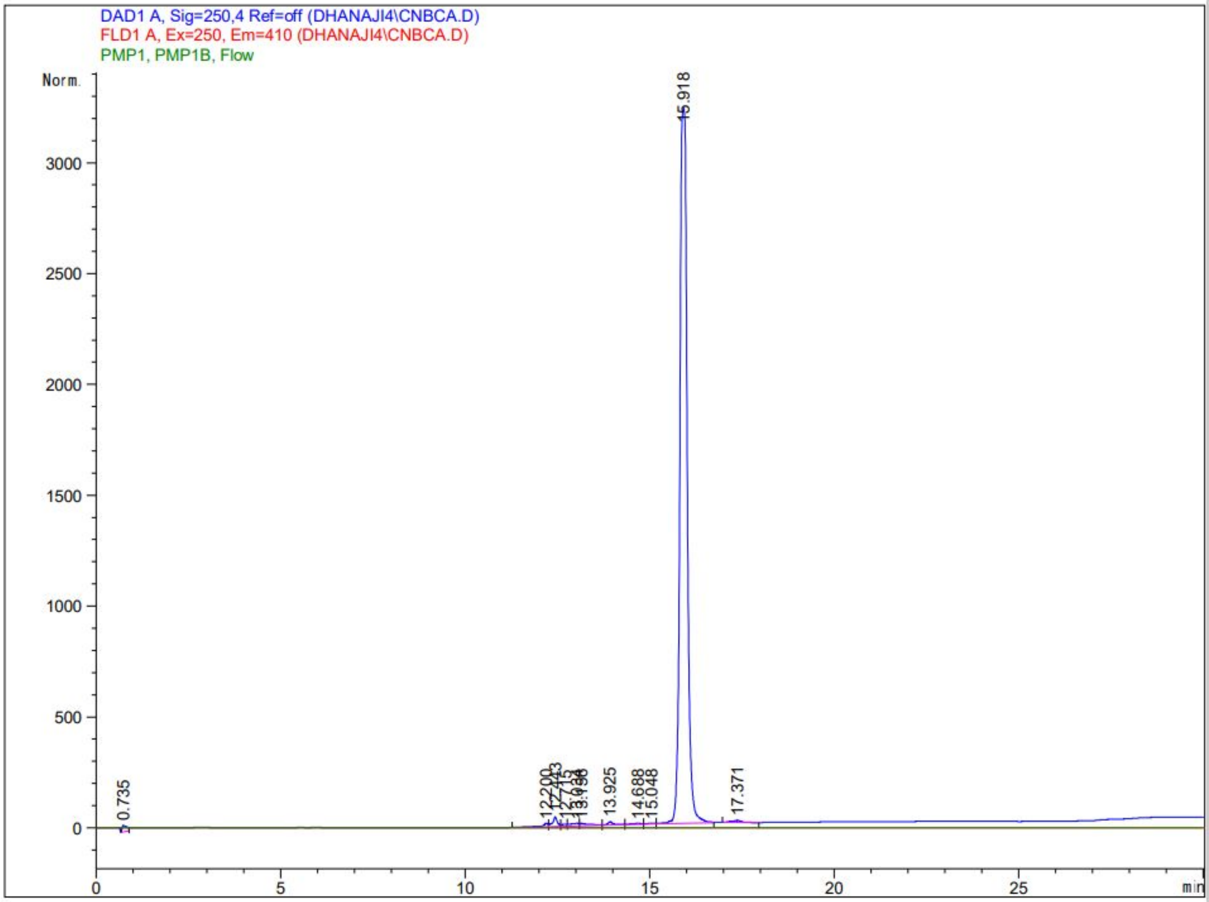

Data File C:\CHEM32\1\DATA\DHANAJI4\CNBCA.D
Sample Name: CNBCA_PURE

===
Area Percent Report
===

Sorted By : Signal
Multiplier: : 1.0000
Dilution: : 1.0000
Use Multiplier & Dilution Factor with ISTDs

Signal 1: DAD1 A, Sig=250,4 Ref=off

Peak #	RetTime [min]	Type	Width [min]	Area [mAU*s]	Height [mAU]	Area %
1	0.735	BV	0.1163	267.00415	30.44949	0.5809
2	12.200	BV	0.1507	176.73354	15.77178	0.3845
3	12.443	VV	0.1243	387.47632	43.35252	0.8430
4	12.715	VV	0.1513	85.85909	8.39896	0.1868
5	13.034	VV	0.2542	187.97073	10.95583	0.4089
6	13.156	VV	0.2568	210.48215	10.64070	0.4579
7	13.925	VB	0.1644	174.27301	14.69181	0.3791
8	14.688	BV	0.2190	92.87885	5.61498	0.2021
9	15.048	VV	0.2044	63.59364	4.40662	0.1383
10	15.918	VB	0.2138	4.41596e4	3224.49878	96.0700
11	17.371	BB	0.2470	160.17509	9.11556	0.3485

Totals : 4.59661e4 3377.89703

Signal 2: FLD1 A, Ex=250, Em=410

===
*** End of Report ***

HPLC_CNBBA

```
=======================================================================
Acq. Operator   : Dhanaji
Acq. Instrument : Instrument 1                    Location : Vial 2
Injection Date  : 3/1/2023 5:37:21 PM
                                               Inj Volume : 100.000 µl
Acq. Method     : C:\CHEM32\1\METHODS\NL1_ANALYSIS.M
Last changed    : 3/1/2023 5:24:15 PM by Dhanaji
Analysis Method : C:\CHEM32\1\METHODS\SRIDHAR-MTI201-AHX-DOTA-2-60-20MIN.M
Last changed    : 3/7/2023 1:08:08 PM by SRIDHAR
                  (modified after loading)
Sample Info     : CNBBA_Final
```

```
Sample-related custom fields:

Name                          |Value
------------------------------|--------------------------------------
Additional Info : Peak(s) manually integrated
```

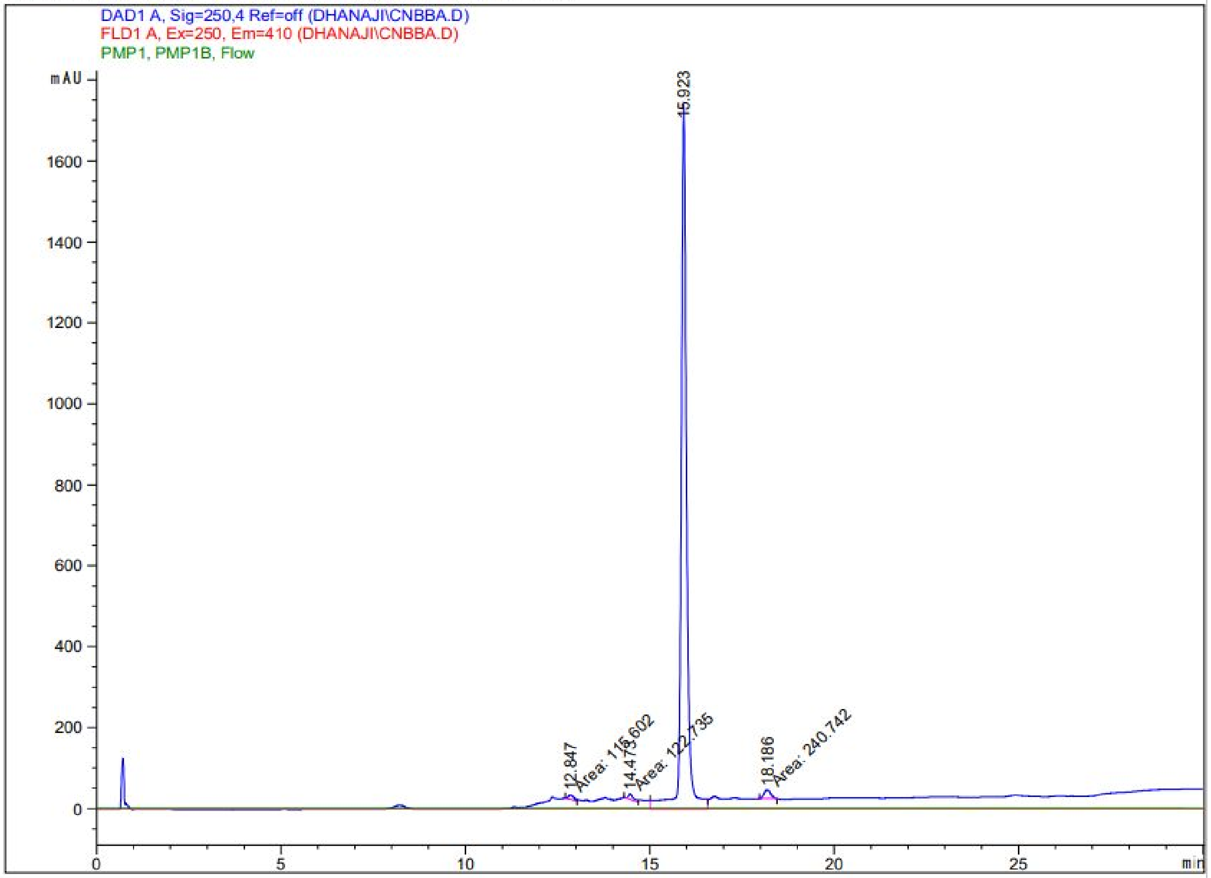

```
Data File C:\CHEM32\1\DATA\DHANAJI\CNBBA.D
Sample Name: CNBBA_FINAL

    =====================================================================
                             Area Percent Report
    =====================================================================

    Sorted By             :      Signal
    Multiplier:                   :      1.0000
    Dilution:                     :      1.0000
    Use Multiplier & Dilution Factor with ISTDs


    Signal 1: DAD1 A, Sig=250,4 Ref=off

    Peak RetTime Type  Width     Area      Height     Area
      #   [min]        [min]   [mAU*s]     [mAU]        %
    ----|-------|----|-------|----------|----------|--------|
       1  12.847 MM    0.1765  115.60183   10.91821   0.6073
       2  14.473 MM    0.1574  122.73482   12.99194   0.6448
       3  15.923 VV    0.1607 1.85558e4 1736.26868  97.4831
       4  18.186 MM    0.1967  240.74246   20.39899   1.2647

    Totals :                   1.90348e4  1780.57782



    Signal 2: FLD1 A, Ex=250, Em=410

    =====================================================================
                             Summed Peaks Report
    =====================================================================

    Signal 1: DAD1 A, Sig=250,4 Ref=off
    Signal 2: FLD1 A, Ex=250, Em=410
    =====================================================================
                          Final Summed Peaks Report
    =====================================================================

    Signal 1: DAD1 A, Sig=250,4 Ref=off
    Signal 2: FLD1 A, Ex=250, Em=410
```
